# Patterns of extreme outlier gene expression suggest an edge of chaos effect in transcriptomic networks

**DOI:** 10.1186/s13059-025-03709-0

**Published:** 2025-09-09

**Authors:** Chen Xie, Sven Künzel, Wenyu Zhang, Cassandra A. Hathaway, Shelley S. Tworoger, Diethard Tautz

**Affiliations:** 1 Department of Evolutionary Genetics, Max-Planck Institute for Evolutionary Biology, Plön, Germany; 2 Biomedical Pioneering Innovation Center, Peking University, Beijing, 100871 China; 3 Shaanxi Key Laboratory of Qinling Ecological Intelligent Monitoring and Protection, School of Ecology and Environment, Northwestern Polytechnical University, Xi’an, 710129 China; 4 Research & Development Institute of Northwestern Polytechnical University in Shenzhen, Shenzhen, 518063 China; 5 Department of Cancer Epidemiology, Moffitt Cancer Center, FL Tampa, USA; 6 Division of Oncological Sciences, Knight Cancer Institute, Oregon Health and Science University, Portland, USA

**Keywords:** Transcriptome analysis, Outlier expression, Mice, Humans, *Drosophila*, Family study, Single-cell analysis

## Abstract

**Background:**

Most RNA-seq datasets harbor genes with extreme expression levels in some samples. Such extreme outliers are usually treated as technical errors and are removed from the data before further statistical analysis. Here we focus on the patterns of such outlier gene expression to investigate whether they provide insights into the underlying biology.

**Results:**

Our study is based on multiple datasets, including data from outbred and inbred mice, GTEx data from humans, data from different *Drosophila* species, and single-nuclei sequencing data from human brain tissues. All show comparable general patterns of outlier gene expression, indicating this as a generalizable biological effect. Different individuals can harbor very different numbers of outlier genes, with some individuals showing extreme numbers in only one out of several organs. Outlier gene expression occurs as part of co-regulatory modules, some of which correspond to known pathways. In a three-generation family analysis in mice, we find that most extreme over-expression is not inherited, but appears to be sporadically generated. Genes encoding prolactin and growth hormone are also among the co-regulated genes with extreme outlier expression, both in mice and humans, for which we include also a longitudinal expression analysis for protein data.

**Conclusions:**

We show that outlier patterns of gene expression are a biological reality occurring universally across tissues and species. Most of the outlier expression is spontaneous and not inherited. We suggest that the outlier patterns reflect edge of chaos effects that are expected for systems of non-linear interactions and feedback loops, such as gene regulatory networks.

**Supplementary Information:**

The online version contains supplementary material available at 10.1186/s13059-025-03709-0.

## Background

Studying gene expression changes through transcriptome analysis has become the standard in unraveling genetic networks, studying cell differentiation and development, exploring patterns of molecular evolution as well as getting insights into genetic diseases. Expression levels are determined through a statistical sampling process of the number of reads obtained in a high-throughput sequencing experiment. Significant differences in expression are estimated based on variance estimates between replicates [1–3]. Variances should ideally follow a particular statistical model, e.g., a Poisson distribution, to properly calculate error probabilities and corrections. However, real data from RNA-Seq experiments usually exhibit overdispersion, which means that the variance of the observed counts is larger than the mean, sometimes due to extreme outlier expression in single or few biological replicates. Overdispersion is usually ascribed to various factors, including technical noise, biological variability, or measurement error. It is therefore common practice to log-transform the primary data, since this leads to a down-weighting of these factors. Since overdispersion affects also statistical power for differential expression analysis, standard analysis programs, such as DESeq2, edgeR, limma-voom, or aFold [1, 3–5], use a negative binomial model with dispersion estimation and adjust the variance of the counts. For most genes this helps to account for the overdispersion and to improve the accuracy and robustness of the differential expression analysis. 

However, it is known that some genes can show extreme expression values in some individuals, which cannot be corrected with overall statistical remedies. Part of the general recommendation for expression data analysis is therefore the removal of such outlier individuals. A standard practice is to identify individuals with extreme expression values, for example in a PCA [6] or a specific denoising pipeline [7, 8]. Such samples are then usually discarded without any further analysis.

These procedures are statistically justifiable under the assumption that outlier values are due to technical error, a concern that was particularly relevant when expression levels were measured via microarray technology. However, with the advent of highly standardized sequencing protocols, the probability of technical error became negligible, obviating even the need to generate technical replicates for quality control [9]. Hence, it is possible that outlier values may have a biological meaning that is underexplored because of standardized pre-filtering steps.

A first dedicated analysis of rare outlier expression was done in human data (the GTEx resource: www.gtexportal.org). It aimed to identify rare genetic variants with possible major effects in individuals [10]. The algorithm to identify these included the condition that the same outlier expression should occur in at least five tissues to ensure that it was actually a genetic polymorphism that had caused them. Many such cases were detected, but many more were again discarded, since they did not conform to the filter criterion.

In the present study, we analyze several large transcriptome datasets from mice, humans, and *Drosophila*, focusing on outlier expression values, even if they occur only in single or few individuals and in single tissues. We show for a sub-dataset that the outliers are fully reproducible in independent sequencing experiments, i.e., should not be considered as technical noise. By using a three-generation family analysis in mice, we show that most of the outlier expression effects are not genetically inherited. We conclude that outlier expression is a biological reality that may be linked to chaos effects that cause sporadic over-activation of transcription of different sets of genes in different individuals.

## Results

The study is based on normalized transcript fragment count data (transcripts per million (TPM) for mice and humans, counts per million (CPM) for *Drosophila*, and normalized counts for snRNA-seq of humans) from population samples. Since we were specifically interested in identifying outlier patterns, we did not log-transform the data and did not exclude any individuals. For the initial analysis, we used whole transcriptome datasets from organs of mice, humans, and *Drosophila* (all sample Lists in Additional file 1: Table S1).

The mouse dataset includes 48 individuals derived from an outbred stock of *M. m. domesticus* originally collected from France (DOM) (Additional file 2: Table S2), as well as between 19 and 20 individuals from outbred stocks of *M. m. musculus* (MUS) (Additional file 3: Table S3), *M. spretus* (SPR) (Additional file 4: Table S4), and *M. spicilegus* (SPI) (Additional file 5: Table S5). Transcriptome data were derived for five organs from each mouse [11]. Further, we used brain transcriptome data from 24 individuals from the mouse inbred strain C57BL/6 (Additional file 6: Table S6).

The human dataset includes 51 individuals for which three organs overlapping with the mouse organs were available in the GTEx dataset [12] plus a subset of 40 of these individuals from which pituitary data were available (Additional file 7: Table S7).

The *Drosophila* dataset included data from two species. The first is from *D. melanogaster*, comprising 27 individuals, for which transcriptomes were separately obtained for head and trunk [13]. The second set included four population samples from *D. simulans*, with 19 to 22 individuals each and transcriptomes obtained from whole flies [14] (Additional file 8: Table S8).

### Identification of outliers

**Fig. 1:**
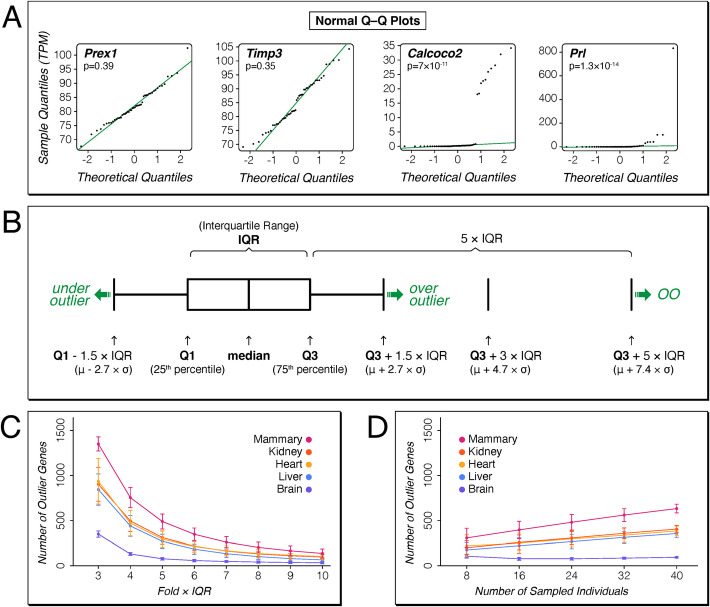
Outlier detection and analysis procedures. **A** Expression data of brains from 48 mouse individuals from an outbred stock (DOM) for four representative genes plotted as Q-Q plots, with the theoretical quantiles on the *X*-axis and the sample quantiles on the *Y*-axis. *P*-values are calculated with the Shapiro–Wilk normality test. There was no significant deviation from the normal distribution for two of the genes (left panels), but a highly significant deviation for the other two genes (right panels). **B** Scheme for using multiples of the interquartile range (IQR) as cutoffs to identify outliers. Under outliers and over outliers are usually called at 1.5 × IQR below or above the first (Q1) or third (Q3) quantile, respectively, which corresponds to 2.7 standard deviations from the mean for normal distribution. We focus our study on over outlier expression, based on Q3 + 5 × IQR, which corresponds to 7.4 standard deviations above the mean for normal distribution. Expression values above this point are denoted as over outliers (OO) throughout, and these are the main focus of the current study. **C** Number of genes with at least one OO at different fold × IQR cutoff levels; averages with standard deviations based on 1000 re-sampling rounds of 24 individuals each from the mouse dataset. **D** Number of genes with at least one OO at the Q3 + 5 × IQR level; averages with standard deviations based on 1000 re-sampling rounds of different numbers of individuals each from the mouse dataset

If the variance in RNA-Seq data would be a simple noise effect, either measurement noise or environmentally induced noise, it should conform to a random distribution model. It is well known that this is not the case for such data. But since we were specifically interested in tracing outlier expression, we started with the noise-driven random distribution model as a null assumption. This can be visualized in quantile–quantile (Q-Q) plots that compare the actual distribution of data points with a normal distribution modeled on these data points. When the data conform to a normal distribution, one will see a straight line and the Shapiro–Wilk normality tests will be *p* > 0.05 (Fig. 1A, left two plots). Indeed, the majority of genes (i.e., on average 72% in our datasets—Table 1) do actually conform to such a model. However, for some genes, there are outlier expression values in some individuals, far away from the Q-Q plot line and the Shapiro–Wilk normality test generates p-values much lower than 0.05 (Fig. 1A, right two plots).

**Table 1:** Summary statistics for the outlier calls for all species and strains

Species	Strain	Tissue		Overall				Per individual		Per gene	
			No. coding genes analyzed	No. individuals analyzed	% genes with random variance*	No. individuals with OO	No. genes with OO	Avg. No. OO genes (SD)	MIN to MAX No. OO genes	Avg. No. of OO (SD)	Max. No. of OO
Mouse	DOM	Brain	12,852	48	85	47	138	5.4 (10.2)	0 to 66	1.9 (1.6)	10
	DOM	Heart	12,249	48	59	45	1112	29.0 (120.9)	0 to 832	1.3 (0.8)	11
	DOM	Kidney	12,211	48	63	48	604	20.1 (41.6)	1 to 264	1.6 (1.3)	10
	DOM	Liver	10,086	48	60	48	862	22.5 (72.5)	1 to 484	1.3 (0.9)	11
	DOM	Mammary	13,114	48	53	48	1069	34.6 (84.5)	1 to 530	1.6 (1.0)	10
	MUS	Brain	12,708	19	91	19	75	5.5 (3.2)	2 to 15	1.4 (0.7)	4
	MUS	Heart	11,261	19	74	18	265	17.4 (26.9)	0 to 123	1.2 (0.6)	4
	MUS	Kidney	11,859	19	80	18	155	9.9 (12.9)	0 to 59	1.2 (0.6)	4
	MUS	Liver	10,008	19	80	19	185	12.6 (6.2)	2 to 30	1.3 (0.6)	3
	MUS	Mammary	12,662	19	73	19	248	15.1 (12.5)	1 to 38	1.2 (0.4)	3
	SPR	Brain	12,630	19	91	19	48	3.1 (1.8)	1 to 7	1.2 (0.6)	4
	SPR	Heart	11,215	19	79	16	284	16.8 (29.7)	0 to 104	1.1 (0.3)	3
	SPR	Kidney	11,877	19	82	19	233	13.7 (19.7)	1 to 83	1.1 (0.4)	4
	SPR	Liver	9982	19	81	19	186	11.1 (12.1)	1 to 47	1.1 (0.4)	3
	SPR	Mammary	12,357	19	72	17	494	27.5 (77.3)	0 to 344	1.1 (0.3)	4
	SPI	Brain	12,689	20	60	16	957	49.6 (163.8)	0 to 716	1.0 (0.2)	4
	SPI	Heart	10,963	20	77	15	220	12.2 (26.9)	0 to 92	1.1 (0.4)	3
	SPI	Kidney	11,464	20	89	14	111	8.2 (14.7)	0 to 47	1.5 (0.8)	3
	SPI	Liver	9799	20	88	14	36	2.2 (2.6)	0 to 11	1.2 (0.7)	4
	SPI	Mammary	11,913	20	82	17	144	9.5 (12.0)	0 to 40	1.3 (0.6)	4
	C57BL/6	Brain	12,308	24	75	16	113	5.5 (15.3)	0 to 76	1.2 (0.5)	4
Human	GTEx	Brain	11,581	51	79	39	738	20.8 (52.4)	0 to 344	1.4 (1.0)	7
	GTEx	Heart	10,691	51	2	42	3065	66.9 (407.0)	0 to 2915	1.1 (0.5)	8
	GTEx	Liver	10,033	51	49	48	1427	38.4 (81.0)	0 to 383	1.4 (0.8)	7
	GTEx	Pituitary	13,451	40	46	39	2102	85.0 (200.3)	0 to 980	1.6 (1.0)	7
Drosophila	D. melanogaster	Head	8200	27	45	27	626	57.3 (87.9)	1 to 245	2.5 (1.8)	6
	D. melanogaster	Body	8102	27	63	27	1006	59.2 (63.8)	31 to 369	1.6 (1.0)	6
	D. simulans_4	Whole Fly	9744	21	88	21	61	3.8 (2.0)	1 to 10	1.3 (0.7)	4
	D. simulans_9	Whole Fly	9750	19	85	17	69	4.8 (6.6)	0 to 31	1.3 (0.5)	3
	D. simulans_27	Whole Fly	9842	22	84	20	103	5.4 (5.6)	0 to18	1.2 (0.4)	3
	D. simulans_28	Whole Fly	9852	19	86	18	55	3.6 (2.8)	0 to 9	1.2 (0.5)	3

* Fraction of genes with Shapiro-Wilk test *p*>0.05 across all individuals in the data set

Here, we are particularly interested in quantifying the numbers of these outlier genes. There are multiple possibilities to generate statistical cutoffs for such outlier detection. One could simply use a cutoff based on multiples of the standard deviation of the distribution. However, this would imply the assumption of a normal distribution of the data, which is not given in the case of multiple outliers, as shown in Fig. 1A. Instead, we use the interquartile ranges (IQR) around the median of the expression values, which are less affected by skewness and extreme values and reflect the dispersion of the data. In this statistic, Tukey’s fences method [15] identifies outliers as data falling below Q1 − *k* × IQR or above Q3 + *k* × IQR, where Q1 and Q3 are 1 st and 3rd quartiles, respectively (Fig. 1B). For reference, when compared to a normal data distribution, *k* = 1.5 would correspond to a 1% cutoff, or 2.7 standard deviations above the mean (*P*-value approx. 0.069). This is usually considered already as very stringent, but not sufficient when very many comparisons are done, as it is typically the case for transcriptome data with thousands of tested genes. It is therefore common to use a *k* = 3, which would correspond to 4.7 standard deviations above the mean (*P*-value approx. 2.6 × 10^−6^), which would satisfy even a stringent Bonferroni correction for multiple testing.

To assess the effect of different k-values on the cutoff for calling outlier values, we used resampling of the mouse dataset of 48 DOM individuals to assess which average percentage of genes would qualify as extreme outliers for different *k*-values in repeated sub-samples of 24 mice from the total dataset. The distribution showed that at *k* = 3, about 3–10% of all genes (~ 350–1350 genes) exhibit extreme outlier expression above the overall expression in at least one individual; these results were similar across different tissues (Fig. 1C). The numbers of extreme outliers continuously declined with increasing k, without a clear cutoff being identifiable (Fig. 1C). For the further analysis, we chose a *k* = 5 (i.e., Q3 + 5 × IQR) as a threshold for very conservatively defining extreme over-expression. In the following, we call expression values above this threshold as “over outliers” (OO), and expression values below the converse threshold (i.e., Q1 − 5 × IQR) as “under outliers” (UO) (compare Fig. 1 B). For comparison, this cutoff corresponds to 7.4 standard deviations above the mean in a normal distribution (*P*-value approx. 1.4 × 10^−13^). Genes that show at least one OO or UO among the sampled individuals are called “outlier genes” in the following.

Given that OOs are mostly found in only one or a few individuals in each dataset, the number of outlier genes is expected to depend on the total number of individuals sampled. We assessed this by random down-sampling from the larger set of 48 DOM individuals. As expected, numbers of genes with extreme expression go down with sample size, but even with only 8 individuals sampled, one can still detect about half of the genes (Fig. 1D). For the following analysis, we focus mostly on datasets with large numbers of individuals (*N* > 40) for the primary analysis.

### Distribution of OOs among individuals

The number of genes with OO expression was systematically determined for each individual for the whole transcriptome datasets according to the above criteria (i.e., the Q3 + 5 × IQR cutoff).

The results are summarized in Table 1 (the detailed data Lists are provided in Additional files 2–8). The majority of individuals sampled carried at least one outlier gene in at least one organ. The overall number of outlier genes per population sample ranged from 36 to 3065, with major differences between species and organs. Major differences were also found at the individual level, with some individuals harboring only a few outlier genes, others very many (averages between 2.2 and 85). In contrast, at the gene level, the average number of OOs per gene is similar across all comparisons (averages from 1.0 to 2.5).

**Fig. 2:**
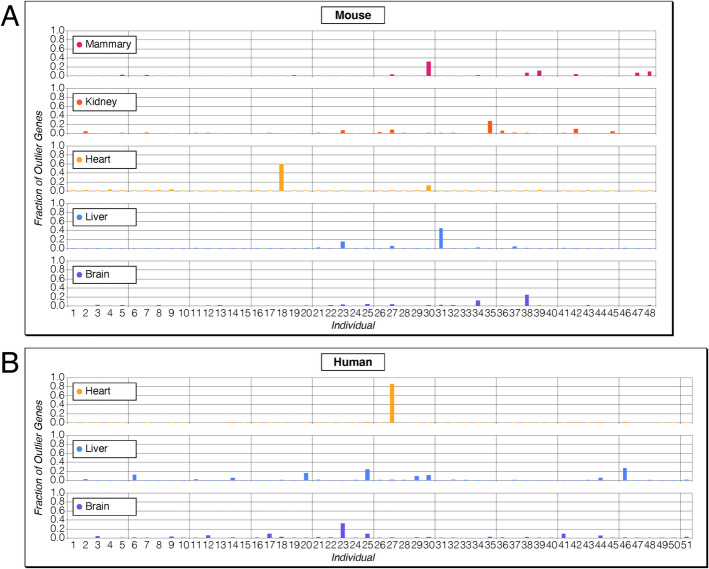
Depiction of the fraction of outlier genes per individual for organs and tissues. The upper panel is for the mouse dataset, the lower panel for the human dataset. The *x*-axis represents the different individuals; the *y*-axis represents the per individual fraction of outlier genes for the given tissue. The highest bars represent “outlier individuals” that show a particularly large number of outlier genes. The data and the numbering of the individuals correspond to the numbering provided in Additional files 2 and 7: Table S2 and Table S7)

The comparison of individuals within any particular sample group shows extreme differences of outlier gene numbers between the individuals. This is evident from the large SD around the average and the large spread between minimum and maximum number of outlier genes per individual (Table 1). In Fig. 2, we visualize this for the mouse DOM data and human data from overlapping organ sets. For example, individual “18” in the mouse data harbors already 832 out of the 1112 outlier genes for the heart (= 75%), but has average numbers of outlier genes for the four other organs. Similarly, individual “27” in humans harbors 2915 out of 3065 of all outlier genes found in the heart (= 95%), but it has only 10 and 22 outlier genes in the brain and the liver. In the following, we denote individuals with particularly many outlier genes as “outlier individuals.”

### Distribution of outlier genes among tissues and species

A subset of genes shows OO expression in more than one organ. In the mouse DOM data, these are 18% (565 out of 3063; Additional file 9: Table S9A) and in the human data 22% (908 out of 4185; (Additional file 9: Table S9H; note that only the three organs with the full set of individuals were included in these comparisons). This shared OO expression between organs could be due to a genetic polymorphism in the respective genes. If this were the case, it should occur in the same individuals for two or more organs, but we find that this is not the case for the majority of them. In Additional file 9: Table S9B (DOM mouse) and Additional file 9: Table S9I (human) we List all outlier genes that show OOs in more than one organ and identify those genes that could be due to genetic polymorphisms since they show outliers in different organs of the same individuals. In the mouse data, we identify 61 out of 565 such genes, and in the human data, 32 out of 908 (all highlighted in yellow in Additional file 9: Tables S9B and S9I). This implies for most OOs that it is unlikely that they are triggered by genetic polymorphisms.

A subset of outlier genes shows OO expression in more than one mouse taxon. Between 7 and 17% of outlier genes in a given organ were found in two or more taxa (Additional file 9: Tables S9C–S9G). When comparing all mouse OO genes found in any organ and taxon with the List of all OO genes found in humans in any of the three common organs, we detected 929 gene pairs of orthologs between mice and humans (Additional file 9: Table S9J).

OOs could potentially also be due to extreme copy number variation in some individuals. In order to assess this, we used a systematic dataset of copy number variants from natural mouse populations [16] and asked whether the corresponding genes show up as OOs. Only four mouse OO genes (*Ppp1r11*, *Rpl27*, *Map3k9*, and *Glo1*) can be associated with known CNV regions in these populations. Therefore, the large majority of OO genes can be considered to be single-copy genes, or genes with only small-scale CNVs that would fall below our expression threshold for calling OOs. Note that we use also a strict filter against reads that could map to paralogues elsewhere in the genome (filtering out genes having at least one paralog with identity larger than 90%—see the “Methods” section), to reduce a possible influence of scattered CNVs through recent gene duplications.

### Technical replication

Given the somewhat scattered occurrence of most OOs, one can ask whether this might be a technical artifact of the library preparation or sequencing reaction. The methodology is generally assumed to be sufficiently robust, in particular compared to the microarray technology [9]. Accordingly, there is a consensus that technical replicates are not required in RNA-Seq experiments. In fact, counting multiple independent sequencing reactions in a given run is already considered to constitute an internal technical replication, which may only generate reproducibility problems when the total number of counts becomes too low, for example, in single-cell sequencing experiments. Similar considerations apply to the library preparation. While this could be biased towards favoring certain sequence types, it should not generate OO patterns for identical RNAs in different individuals.

To verify these general assumptions, we generated for five individuals two separate sequencing libraries from the same brain RNAs (from the pedigree animals described below), but using different kits and sequenced the libraries on different instruments (Illumina NovaSeq and NextSeq). A total of 17 outlier genes were found in these five animals, in different combinations. Each was faithfully detected as OO in both replicates, even those with relatively low TPM values overall (Additional file 10: Table S10). Hence, although this was only a small replication experiment, it generated no indication that OO calls could be artefacts of a given library preparation or sequencing instrument. In fact, the results from the repeated finding of the same outlier genes in different organs and species (see above), as well as the family analysis and the co-expression modules described below, support further the notion that the expression measurements via RNA-Seq are a faithful method to trace OOs.

### OO expression in an inbred strain

Given that the data above suggest that OO expression could be a spontaneous phenomenon, independent of genetic polymorphisms, we asked whether we would observe OOs also in transcriptome data from an inbred mouse strain. To assess this, we obtained brain transcriptome data from 24 individuals of the isogenic lab strain C57BL/6 (Additional file 6: Table S6). 16 of these showed an OO expression, involving 113 genes (Table 1). Most interestingly, among the C57BL/6 Mice, we also found an outlier individual with 76 outlier genes. Although a small number of polymorphisms segregate also in inbred mouse strains [17], the fact that the average numbers of outlier genes in the inbred mice are similar to the outbred mice supports the notion that DNA polymorphisms are not the main drivers of OO expression.

### Inheritance patterns of OOs in pedigree data

**Fig. 3:**
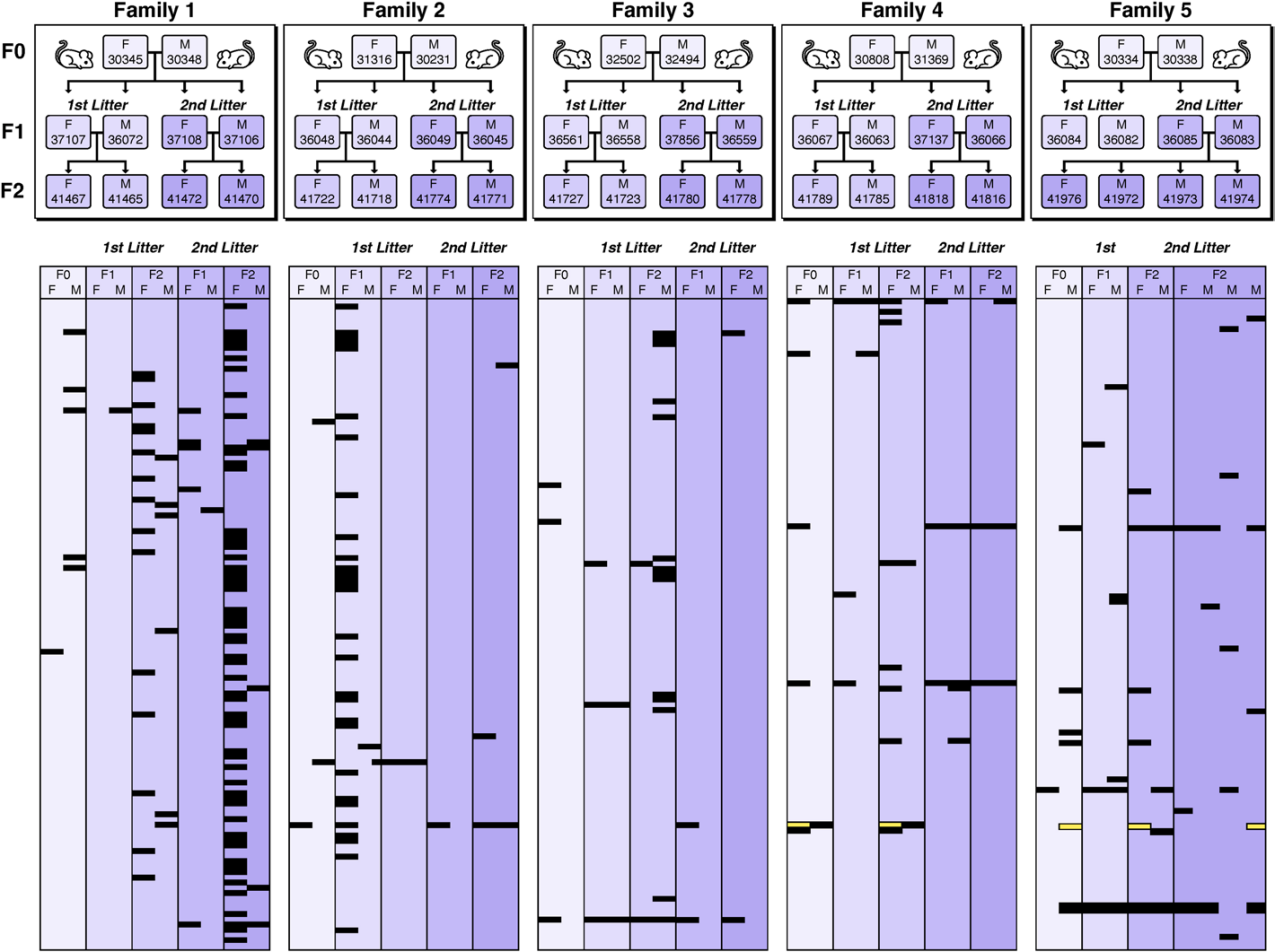
Depiction of the five mouse family pedigrees and OO patterns for brain. The upper panels show the pedigrees, whereby families 1–4 have the same scheme (see also overview in Additional file 11: Table S11A). In family 5, the F1 of the first litter had no offspring. To compensate for this, we used four offspring of the F1 of the second litter. The lower panels depict rows for each gene that showed an OO in at least one individual. The OOs are highlighted in black; yellow indicates under outliers (UO—note that genes that showed only UOs are not included). The parental columns are always first in each block, followed by two F1 and two F2 individuals for each Litter. TPM values are available in Additional file 11: Tables S11B–S11D

To further test whether genetic polymorphisms, or inherited epigenetic signals could be a key driver of OO expression, we set up five three-generation families of an outbred stock of *M. m. domesticus* originally collected from Germany. The breeding schemes are shown on the top of Fig. 3 and in Additional file 11: Table S11A). Brain, kidney, and liver transcriptomes were generated for each individual and analyzed as described above. The distribution of the OO patterns across the family structures is shown in Fig. 3 for the brain data; the data for all three organs are provided in Additional file 11: Tables S11B–S11D. We identified a total of 123 (brain), 178 (kidney), and 659 (liver) outlier genes in the total dataset of 50 individuals, which is in the range of the number of outlier genes found for the first set of DOM individuals shown in Table 1.

Expression levels of genes should normally add up between the two alleles when they are under cis-regulation control [18]. This will show up as an additive (co-dominant) inheritance. In the case of trans-regulation, it is a question of the presence or absence of the trans-regulator, which will also show up as a dominant inheritance pattern. In both cases, one does not expect a recessive pattern. Based on these considerations, we checked whether the distribution of OOs in the families was compatible with a Mendelian dominant (or co-dominant) inheritance. We classified all genes into *Mendelian (M)*, when the inheritance patterns of the OOs were compatible with Mendelian inheritance in at least one family and as *Spontaneous (S)*, when OO turned up in offspring, but not in their parents. We also used two further classifications where inheritance assignment was not possible (*P* for *Parental*, i.e., only occurring in the F0 and *Q* for *Questionable*, i.e., not clearly resolvable). The classifications are based on an automated algorithm and were combined with a manual analysis according to the criteria detailed in the Methods section.

In this gene-by-gene analysis, we found that the majority of OOs could be classified as having spontaneously arisen (brain 101 *S* vs. 10 M, kidney 87 *S* vs. 15 M, Liver 158 *S* vs 17 M) (Additional file 11: Table S11B-D). For the *M* genes that were expressed in more than one organ, we found that the OO pattern was usually found in the same individuals of the other organ (Additional file 11: Table S11E), confirming the notion of a genetic polymorphism driving the pattern.

An interesting *Q-classified* gene in the brain data is *Glyoxylase 1* (*Glo1)*. This is a gene known to have high copy-number variability in the *M. m. domesticus* populations [16] and its expression level is known to influence anxiety patterns [19]. It shows variable expression levels among the individuals, of which only some may be inherited. It is also the only gene that shows both, OO as well as under-expression (under outlier, UO) (Fig. 3 and Additional file 11: Table S11B). This suggests that new copy number variants are frequently generated in these family pedigrees. A second Q-classified CNV gene (*Rnase2a*—alternative name *Ear11*) occurs in the Liver data, but only in two individuals of family 5. The liver data include also the CNV gene *Hamp2*, but only in a single individual. Hence, for the latter two genes it is unclear whether their CNV status results in outlier expression.

There are also at least four individuals in the family set which can be classified as “outlier individuals,” based on harboring particularly many outlier genes. Figure 3 shows this for two individuals (individual 41,472 in family 1, F2, second Litter and individual 36,048 in Family 2, F1, first litter). In both cases, the parents did not have large numbers of OOs, suggesting that the status as “outlier individual” has arisen spontaneously in the respective offspring. In the liver data, there are two parental F0 animals in family 1 and family 2 (individuals 30,345 and 31,316—Additional file 11: Table S11D) that constitute such “outlier individuals.” Both did not inherit this status to the F1 in the respective families, which is also compatible with the assumption that the status as “outlier individual” is a spontaneous one.

### Correlated OO expression

**Fig. 4:**
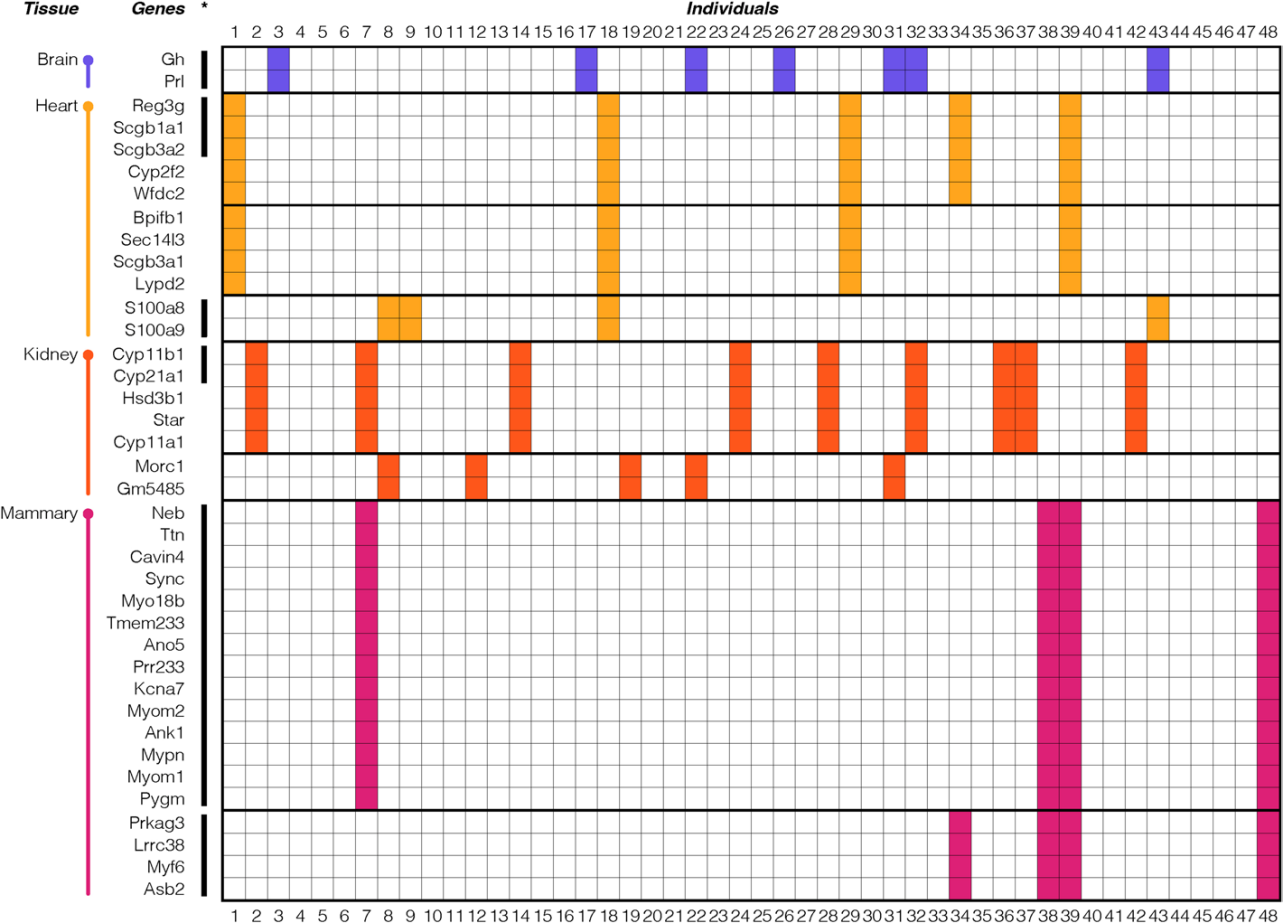
Correlated OO expression in gene groups in mouse organs. Genes are shown for which the pairwise comparisons revealed at least four individuals with OOs and no individual gene with OO expression in another mouse (an extended version of this figure with a Minimum of three individuals sharing OO expression is provided in Additional file 12: Table S12A). OO expression is marked by colored shading. Note that liver shows no such gene pairs or gene groups, and thus is not included. Black lines to the left indicate co-expression groups that are still visible when the outlier individuals are removed from the respective comparisons

During the analysis of the data, we noted for some gene sets a correlated OO expression in their respective tissue. To assess this systematically, we focused on genes in which at least three individuals shared the OO expression for two or more genes, and no other individual showed an OO expression for the respective genes. This is a rather strict requirement, but even under this condition, we find many such correlated expression groups. As an example, we show in Fig. 4 a subset of the groups and distributions between individuals for the mouse organ data. This represents the cases where at least four individuals share an OO expression; the extended version, with at least three individuals sharing an OO expression, is provided in Additional file 12: Table S12A.

OOs in Fig. 4 are marked by colored shading (colors according to tissues), outlier gene groups by boxes. In the heart, there is the gene pair of S100 calcium binding proteins A8 and A9 (*S100a8*/*S100a9*), which is located in the same chromosome region, but on different strands. The genes code for highly studied Ca-binding proteins, with a role in inflammation and cancer [20]. They act usually as heterodimers; a correlated expression is expected for them.

Heart includes further a group of five outlier genes, of which two are members of a family of Secretoglobins on different chromosomes (*Scgb3a2* and *Scgb1a1*), one a member of the Cytochrome P450 family of proteins (*Cyp2f2*), one a member of the Regenerating islet-derived gene family (*Reg3g*) and the WAP four-disulfide core domain 2 gene (*Wfdc2*). In kidney, there is also a group of five outlier genes, three of them part of the Cytochrome P450 family (*Cyp21a1*, *Cyp11b1*, and *Cyp11a1*, all encoded on different chromosomes), in conjunction with a Hydroxy-delta-5-steroid dehydrogenase gene family member (*Hsd3b1*) and the Steroidogenic acute regulatory protein (*Star*). To assess whether these groups may have joint functions, we used GO analysis via PANTHER [21], but without significant results. On the other hand, the top block of 14 co-regulated genes in mammary are enriched for genes involved in myofibril assembly, including Myomesin 1 and 2 (*Myom1*, *Myom2*), Myopalladin (*Mypn*), Caveolae-associated protein 4 (*Cavin4*), Titin (*Ttn*), and Nebulin (*Neb*).

Note that the group modules show frequently partial sharing of individuals with other group modules (e.g., visible in heart and mammary in Fig. 4, and more extensively visible in the extended supplementary Tables with the full data for mice, humans and *Drosophila*—Additional file 12: Tables S12A–S12C).

We asked whether these co-regulated outlier gene groups reflect modules that are also co-regulated when they include no individuals with OO expression. To assess this systematically, we removed the individuals with OO from the data and asked whether the remaining gene expression levels between the individuals was significantly correlated (Kendall’s tau for every combination of gene pair, *P* < 0.05 with Bonferroni multiple test correction). It is indeed possible to find correlated gene groups in this way which overlap at least partially with the gene modules identified via over-expression analysis. These are marked by black lines in Fig. 4. Interestingly, the whole gene group of 14 genes in mammary that may be involved in myofibril assembly shows also up as a co-regulated group when OOs are removed.

Other correlation groups are only partly visible when the OOs are removed, e.g., the two groups of five genes discussed above for heart and kidney (Fig. 4), or even absent (compare also extended data in Additional file 12: Tables S12A–C). In the *Drosophila* data, there are very large and complex groups of outlier genes with sharing between individuals, especially in the head, but very few of them show co-regulation when the OOs are removed (Additional file 12: Table S12C).

We conclude from this analysis that outlier genes that are co-expressed in multiple individuals identify to some degree normal co-expression modules in cells, which may, or may not be detected by other co-expression analyses as well.

### Outlier in single cell data

Gene expression is generally known to be pulsatile, whereby only a subset of cells in a tissue show high expression at a given time [22, 23]. We have therefore also analyzed single-cell data to trace the phenomenon of over-expression in some individuals. However, because one needs the comparison between multiple individuals, there are still only a few datasets that can be used for this purpose. We have chosen data from an Alzheimer study, which has investigated two human brain regions (DFLPC and MTG) from many individuals using single-nucleus transcriptomics [24]. We include a total of 53 individuals and up to 15 cell types that were consistently identified between them (suppl Table S1F), each covered by at least 100 cells per individual. Given that the sequencing depth at each single cell is relatively low, we used first the mean across all cells for the given cell type in a given individual for the overall analysis.

**Fig. 5:**
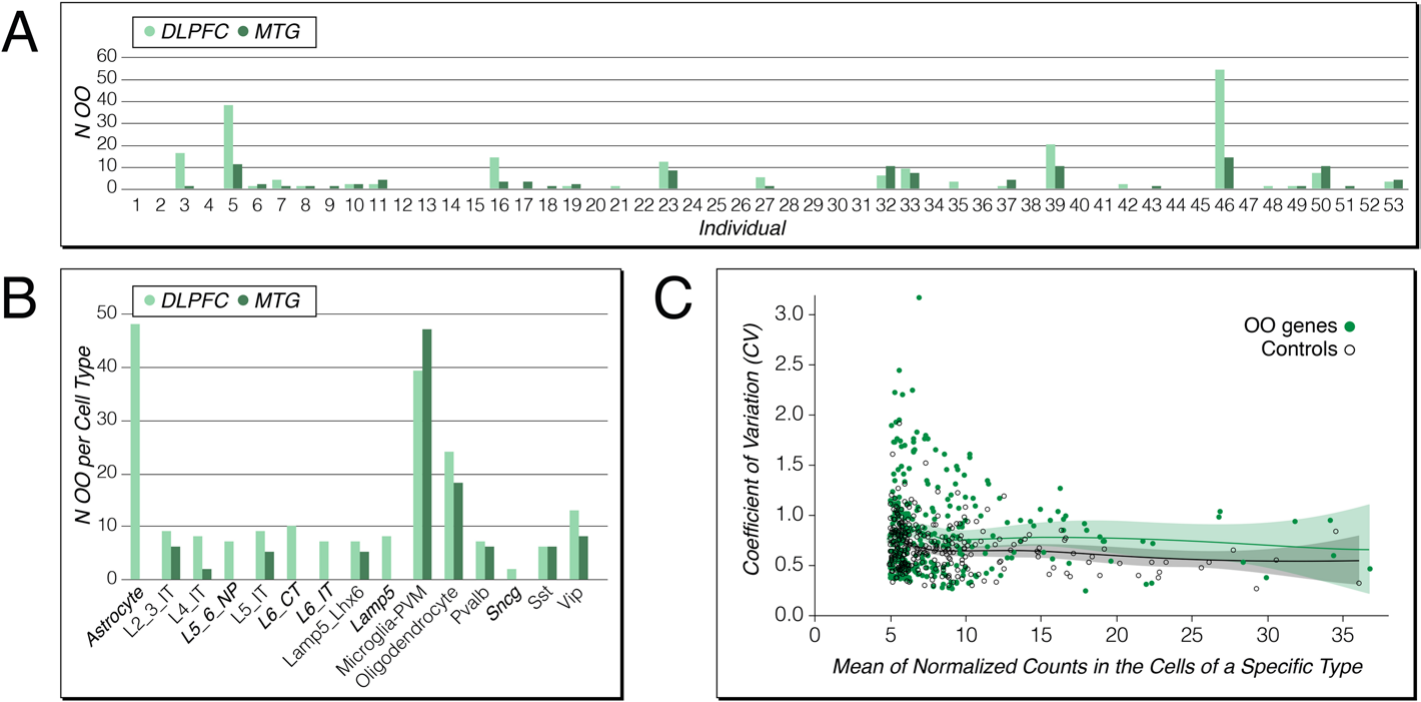
Numbers of OOs in individuals and cell types and spread of variances of OO genes in single nuclei expression data. **A** Cumulative numbers of OOs for the cell types from each of the two sampled tissues, for all individuals. **B** Number of OOs per cell type. Note that MTG includes only a subset of the cell types of DLPFC (Additional file 13: Table S13A); the cell types specific for DFLPC are written in italics. **C** Spread of variances of OO genes compared to control genes. Every OO gene is matched with a control gene from the same cells, which is expressed at a similar level, but without much variance between the individuals. The two distributions are significantly different (*P* = 1.2 × 10^−6^, Wilcoxon signed rank test)

The overall analysis is comparable to the data from the organs described above. We found between 0 to 68 OOs per individual (average 5.8, SD = 12.5; 0 to 26 outlier genes, average 3.4, SD = 5.9) and 2 to 48 OOs per cell type (1 to 40 outlier genes) (Additional file 13: Table S13A). The numbers between the individuals differ substantially, and the two brain regions show different numbers for the same individuals (Fig. 5A). There are also major differences in OO genes between the cell types, whereby the numbers are similar for those cell types that occurred in both brain regions (Fig. 5B).

However, the power of the single-cell data Lies in asking whether the OO status across the mean of the transcript counts is generated more or less by all cells, or by a subset of particularly highly expressing cells. If only a subset of cells of a given cell type shows outlier expression, one would expect a higher variance in expression for outlier genes compared to a control set of genes. To test for this, we focused on all 145 genes that showed an OO in at least one individual and in one or more cell types. We asked whether the variance in expression in the individual cells for a given cell type would exceed the variance of a control gene in the same set of cells. Control genes were chosen such that they had a similar overall expression level as the OO gene, but a variance within the Q1-Q3 range across individuals. Given that a number of the 145 genes were expressed as OO in more than one cell type, we could do a total of 307 such comparisons (Additional file 13: Table S13B). We found that although the overall variances (measured as coefficient of variation = CV) was significantly higher across the OO genes than across the control genes (*P* = 1.2 × 10^−6^, Wilcoxon signed rank test), there are substantial overlaps (Fig. 5C). In fact, only 176 comparisons have a higher CV, 131 a lower one (Additional file 13: Table S13B).

These results suggest that there is an inherent tendency for the expression of outlier genes in individual cells to be more heterogeneous, but not for all outlier genes. It seems clearly not the case that OO expression is generally due to pulsatile gene expression in subsets of individual cells, since this would have shown up as consistently higher CVs compared to control genes for all comparisons. Instead, the fact that some outlier genes show even lower CV implies that cell types as a whole can become subject to outlier expression at a given time (i.e., the time of sampling the respective individual).

### Search for epigenetic signatures of outlier expression

One would expect that outlier expression should become visible in epigenetic signatures of gene expression, e.g., methylation patterns, openness of chromatin or polymerase loading. The EN-TEx project [25] has generated assorted epigenome data on four individuals of the GTEx samples. We have analyzed these data to assess whether there is any obvious epigenetic pattern for OO expression. We analyzed ATAC-seq and DNase-seq to assess promoter openness, DNAme array data for methylation states at promoters, POLR2A ChIP-seq for polymerase loading, and histone ChIP-seq to determine histone modification states around promoters. Across the data from nine organs, we found 13 genes that had an outlier status that could be compared to corresponding data from an individual that showed no outlier expression. However, no clear consistent effect could be detected in this limited set. Only one gene (*APOA1*) showed a much higher polymerase loading (Additional file 14: Fig. S1), but this was not seen for the other genes.

At the tissue level, multiomics data of human dorsolateral prefrontal cortex samples are available in the “Rush Alzheimer’s Disease Study” in ENCODE [26]. We chose 45 individuals with RNA-Seq, DNase-Seq, CTCF ChIP-Seq, H3K4me3 ChIP-Seq, H3K27ac ChIP-Seq, and H3K27me3 ChIP-Seq data from the same samples (Additional file 1: Table S1G). DNase-Seq indicates the relative openness of promoters, CTCF signals indicate the binding of the zinc finger transcription factor CTCF (but this is not expected to correlate with transcription rate), H3K4me3 and H3K27ac are active transcription markers, and H3K27me3 is a repressive transcription marker. We retrieved for all genes that showed at least one OO the corresponding values for the chromatin marks derived from the same samples from which also the RNA-Seq data were derived. This resulted in a List of 165 genes. For this set of genes, the correlations between the RNA expression values (TPM) and chromatin mark values were rather poor: 0.12 for DNase, 0.04 for CTCF, 0.07 for H3K4me3, 0.10 for H3K27ac, 0.00 for H3K27me3 at promoter regions, and −0.02 for H3K27me3 at gene body regions. Hence, the chromatin marks have only a very poor predictive value for the transcription levels and thus also for the correlation with OO values. Still, we find for some genes that the individual with an OO in a given gene has also the highest signal for one of the activating chromatin marks (details included in Additional file 15: Table S14) and visual representation in Additional file S16: Fig. S2). The fraction of such genes is similar to the respective overall correlation coefficients, but given the small numbers, this could be a random effect. For the repressive chromatin mark H3K27me3, we find for three genes that the OO individual carries also the highest H3K27me3 score, implying at least for these genes that the inhibiting mark does not prevent OO status.

Overall, with the currently available data, the analysis of epigenetic marks in the context of OO expression does not point to a consistent correlation. It seems possible that only gene-specific and cell-type-specific effects can eventually be traced with more data.

### Prolactin and growth hormone

**Fig. 6:**
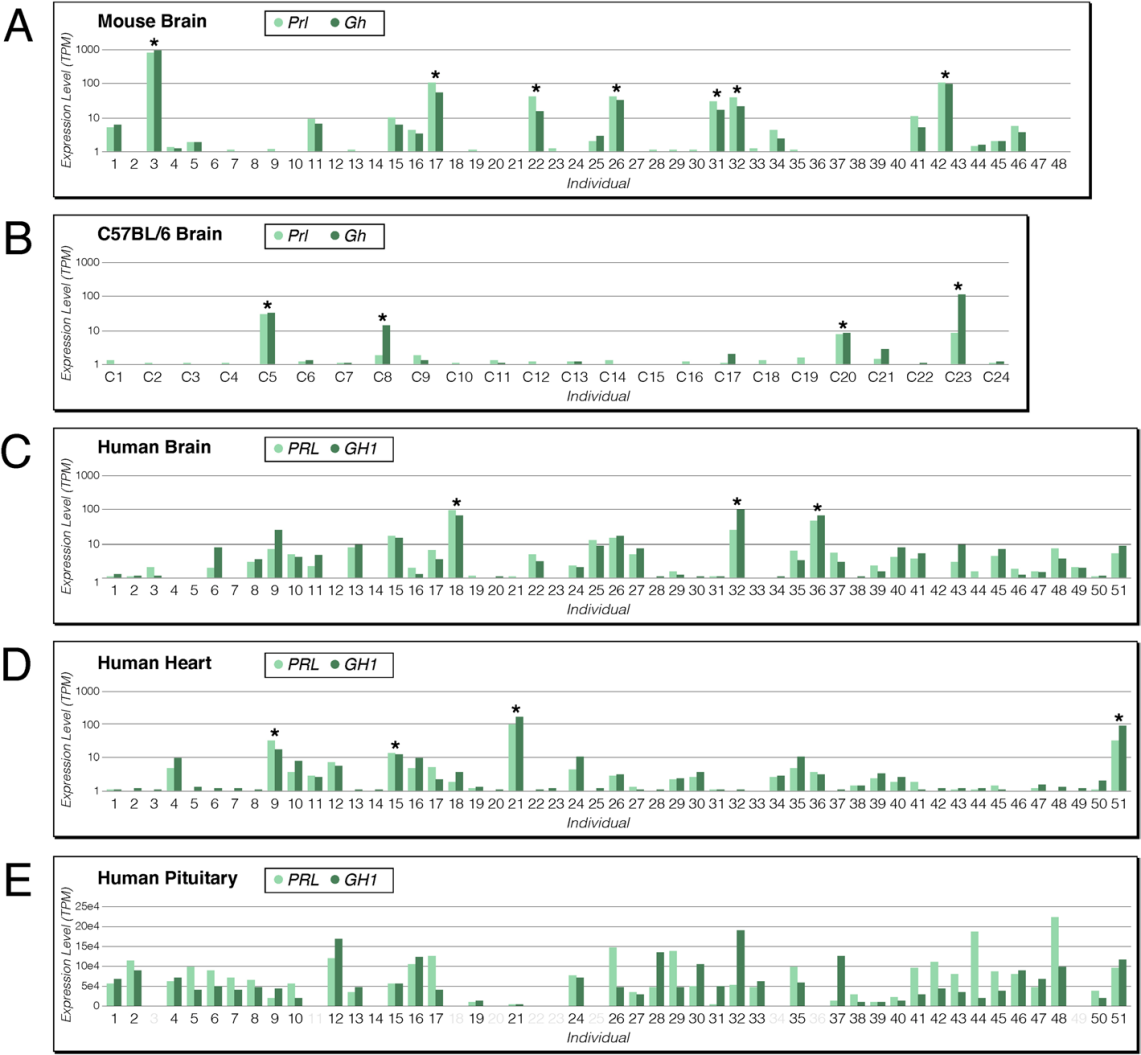
Expression levels of prolactin (light green) and growth hormone (dark green) transcripts between individuals. Data include mice (**A** and **B**) and humans (**C** to **E**). The *Y*-axis for (**A**–**D**) is on a log scale of “TPM + 1”. **A** DOM wild mouse brain, (B) isogenic mouse strain C57BL/6 brain, **C** human brain, **D** human heart, and **E** human pituitary gland. OOs meeting the Q3 + IQR * 5 threshold are marked with stars. Note that the panels for humans relate to the same individuals (51 for brain and heart, 40 for pituitary gland); OOs occur in different individuals for brain and heart

Among the outlier genes shared between mice and humans are the hormone genes, prolactin (*Prl*/*PRL* in humans) and growth hormone (*Gh*/*GH1* in humans). They belong to the co-regulated gene pairs described above, with the same individuals displaying the OO expression status for both genes in the outbred and inbred mice, as well as in humans (Fig. 6). In humans, both genes are also expressed in the heart, and they show also OOs for this tissue, but in different individuals compared to the brain (Fig. 6C, D). *Gh* and *Prl* expression are also significantly correlated when the OO individuals are removed, both in the mouse brain (Fig. 4, top) as well as in the human brain and heart (Additional file 12: Table S12B). Hence, the OO expression of this gene pair reflects an inherent co-expression of these genes in the respective tissues.

However, the expression of these hormone genes in the general brain and heart tissues is relatively low and of unknown function. The main expression of the genes occurs in the pituitary gland, but in different cell types, the somatotroph and lactotroph cells, respectively. We have therefore also examined the GTEx data for the human pituitary gland from the subset of individuals that overlap with the main human GTEx dataset described above. The analysis is included in Table 1.

While the human pituitary gland shows generally a large number of outlier genes, *PRL* and *GH1* are not among them. However, there are substantial expression differences between the individuals (Fig. 6E), implying a large IQR spread, such that some highly expressing individuals (e.g., individuals 44 and 48 for *PRL*, or individual 32 for *GH1*) fall below the chosen cutoff. Interestingly, there is also no correlation between *PRL* and *GH1* expression levels in the pituitary gland (Fig. 6E), as is to be expected given that they are expressed in different cell types of the gland.

Hence, the expression characteristics of the two hormone genes in the secondary tissues (brain and heart) are different from the primary tissue (pituitary gland). This implies that the overexpression in the secondary tissues is not simply the consequence of using the same promoter signals as in the primary tissue. Of note, *Prl* and *Gh* showed also up as OO in the mouse family brain data (Additional file 11: Table S11B), both in the same four individuals, *Gh* in one additional individual. In three out of the five cases, there was no parental individual from which the expression could have been inherited, i.e., these would be classified as spontaneous activation (*S*). However, in Family 5, Litter 2, an inheritance could not be excluded. Hence both genes were conservatively classified as *Q* (Additional file 11: Table S11B), although *S*, as is suggested by the OO pattern in the inbred mouse strain (Fig. 6B), is also compatible.

### Outlier in hormone level measurements

All the above data are from RNA measurements, which represent only a single point in the lifetime of the individual. It is therefore not possible to determine whether outlier expression status is a short-term phenomenon (in the order of days or weeks) or whether it is stable for a given individual over the lifetime. This is different for hormone measures, which can be done repeatedly for the same individuals.

Prolactin and growth hormone are often measured in large human cohorts. A recurrent observation in these studies is the identification of individuals with exceptionally high hormone levels [27, 28]. While high hormone levels might be caused by a medical condition [29], we analyzed the data under the aspect of OO expression patterns reflected at the protein level.

We evaluated plasma concentrations of prolactin and growth hormone measured as part of case–control studies for breast cancer nested within two large prospective cohort studies, the Nurses’ Health Studies (NHS/NHSII). Details of assay measurements and quality control are available in [30, 31]. For outlier status, we used the Q3 + 3 × IQR cutoff since the multiple testing problem is not an issue here (only two hormones tested, in contrast to thousands of genes in the RNA analysis above) and since the Q3 + 5 × IQR cutoff appeared to be too stringent for the protein data.

**Fig. 7:**
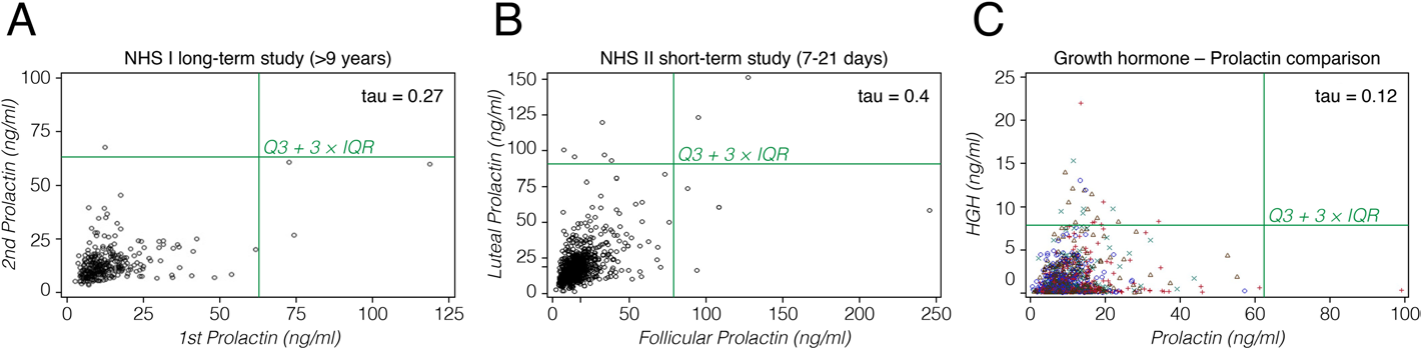
Hormone expression level comparisons in humans. Results are shown for prolactin measurements at two different times (**A**, **B**), and a comparison between prolactin and growth hormone in the same individuals **C**. **A** Data from 310 females for which measurements were taken between 9.5 and 15.25 years apart. **B** Data from 974 females for which measurements were taken during one menstrual cycle, the first in the follicular phase, the second in the luteal phase. **C** Data from 1200 females, taken at different ages for prolactin and growth hormone (HGH). Different symbols represent different ages that are not further considered here

In one study (NHS), data were obtained from 310 females, with two measurements of prolactin approximately 9–15 years apart (first blood sample collected from 1989 to 1990 and second from 2000–2003). The overall correlation between the two measurements was moderate but significant (0.27; Kendall’s tau; *p* < 0.0001). Four individuals showed outlier levels, three in the first measurement, one in the second, but none showed outlier levels at both times (Fig. 7A).

In the second study (NHSII), 974 females were tested during a menstrual cycle, with the first measurement in the early follicular phase (about 5 days after onset of menses) and the second in the luteal phase, i.e., about 7–9 days before the anticipated start of the next period; the samples were collected within the same cycle (within the same month). This tests for short-term stability of outlier levels. The correlation within person for these values was higher than for the long-term study (0.4; Kendall’s tau; *p* < 0.0001). Eleven individuals with outlier levels were detected, four in the follicular phase only, five in the luteal phase only, and two in both phases. Individuals with outlier levels in one phase generally showed elevated hormone levels at the other stage, although below the cutoff (Fig. 7B). This result suggests that outlier expression could persist for a few weeks, possibly with a peak for a short time and declining levels afterwards.

In NHS/NHSII, a subset of women with prolactin measures also had growth hormone measures on the same blood sample, such that we could assess the correlation between them. In the NHS data, 22 individuals with outlier levels were detected for growth hormone among 1200 females in NHS, but only one individual with outlier level for prolactin, and this individual did not show an elevated growth hormone level (Fig. 7C). Nonetheless, there was still a low, but significant overall correlation across all data (0.12; Kendall’s tau; *P* < 0.0001).

To put this into context with the RNA data of pituitary glands, we analyzed all 283 individuals from the GTEx study. In this larger group and with a Q3 + 3 × IQR cutoff, three individuals had PRL with an OO, but still none had one for GH1. The overall correlation across all data was similar to the protein data (0.12; Kendall’s tau; *P* = 0.0021).

These observations show that the strongly correlated patterns of OO expression seen for the two hormones in secondary tissues are not reflected in the circulating hormone levels. On the other hand, the RNA expression patterns from the primary tissue (the pituitary gland) are well compatible with the protein data.

## Discussion

The occurrence of outliers in gene expression values is a well-known phenomenon in transcriptome data. However, this has so far been treated as a noise component of the data, and it was corrected by filtering techniques, such as removal of certain samples or transformation and/or modeling of the data spread to reduce the relative impact of outliers on the overall analysis.

Here we have studied RNA expression across multiple species, including mice, humans, and *Drosophila* to better understand the occurrence and patterns of outlier expression. We use an interquartile range (IQR) statistic without data transformation to identify extreme over-outlier (OO) in the datasets that are far beyond expected statistical fluctuations. We show that the detection of such outliers is reproducible between different library preparation protocols and different sequencing machines.

We find that OOs are distributed unevenly across individuals, with hundreds to thousands of genes with an OO in one or a few individuals in a dataset. Some individuals harbor particularly many outlier genes in a given organ but not in another organ from the same individual. Most of these outliers are not driven by genetic polymorphisms or copy number variation, as seen in both inbred mouse strains and a three-generation family study in mice.

OOs can occur in co-regulated modules, in the sense that they are co-expressed in multiple individuals. Several of these modules reflect also a co-expression of the same genes when they are expressed in a normal range. However, some modules show up only in the outlier gene expression, but not in the normal expression. This effect is particularly evident in the *Drosophila* data.

### Prolactin and growth hormone gene expression

Among the co-expressed genes with frequent OO patterns in mice and humans are the hormone genes prolactin and growth hormone. While the highest expression of these hormones is in the pituitary gland, they are also expressed in multiple other tissues, including the brain cortex and the heart, but at much lower overall levels. In the mouse brain preparations, the pituitary gland is included in the whole brain. But given that it is so small, the RNA derived from it would be highly diluted by the RNA from the other brain areas. Hence, we assume that the expression that we can detect in mouse is mostly from the general brain tissue. For the humans, we could separately analyze the pituitary gland and it harbors indeed many genes with OO patterns. *PRL* and *GH1* expression is approx. 10,000 times higher in this tissue than in any other tissue, i.e., they show already extreme expression in their normal function. Accordingly, it is more difficult to trace a much higher OO level. Indeed, both of these genes did not show an OO pattern in any of the 40 analyzed individuals, at least for our strict cutoff criteria. Still, there is major variation in expression of these genes between individuals; hence, the general effect that leads to OO patterns could still be active. Interestingly, there is only a weak correlation in expression levels of *PRL* and *GH1* in the pituitary gland, in contrast to a strong correlation in the brain cortex and the heart. This suggests that the regulatory input into these genes is different between the peripheral tissues and the main tissue of expression. On the other hand, the expression correlation in secondary tissues is conserved between mice and humans, implying that it is not a chance effect. These hormones have rather pleiotropic functions across the body and in various physiological processes [32] and additional functions in tissues where they are co-expressed at low levels seem possible.

### Longitudinal analysis

The longitudinal analysis of the prolactin hormone data in humans suggests that the OO expression may be somewhat stable within a matter of days or weeks. However, currently this can only be a first attempt to approach this question. Given that the RNA expression in the pituitary gland did actually not show up prolactin to be an OO gene, we cannot even be certain that the large variation of the prolactin expression is actually based on the same principles as the OO expression. High prolactin expression may also be caused by adenomas [29].

Ideally, longitudinal studies should be done based on RNA-Seq experiments to screen all expressed genes in parallel. There are some first developments to establish such a noninvasive sampling for RNA expression [33]. Such approaches will allow setting up dedicated studies to assess how long a spontaneous OO expression lasts.

### Expression modules

Several sets of OO genes are co-expressed in multiple individuals, which serves actually as additional evidence that this is not a simple noise effect. Some of these co-expression groups would be considered as gene modules in standard correlation analyses, since they are also co-expressed when the OO individuals are removed. Interestingly, most modules of OO genes are broken up between the individuals, i.e., they occur in different, but overlapping combinations. This could be interpreted as rather short-lived phases of the OO status, which might end at different times in different individuals.

### Spontaneous activation of high-expression promoters?

One could speculate that the OO incidences in given tissues are due to accidental activation of regulatory pathways that trigger a high expression of these genes in other tissues. This would predict that OO is primarily observed in tissues that have a low expression of a gene, which has a very high expression in another tissue. This would be akin to the prolactin growth hormone genes discussed above, although their co-expression in the peripheral tissues does not really support such an interpretation. Still, we tested this interpretation across all genes.

In humans, we used the List of median expression values of all genes across all 54 GTEx tissues to ask whether OO expression is more Likely in tissues where they are not maximally expressed. In the Liver, there are 21 OO genes that have also normally the highest expression in liver, which reflects approximately the proportion of all genes that have the highest expression in the liver (1.4% vs. 2.2%). On the other hand, heart and brain have only one OO gene each that is also expressed highest in these tissues, which is a lower fraction than expected overall (heart: 0.03% vs 0.4% and brain: 0.13% vs 0.4%). So again, the answer seems equivocal at this stage.

### Relationship to pulsatile gene expression

It is well established that expression of a gene in a tissue does usually not occur in all cells at the same time, but only in a subset of cells with particularly high expression pulses [22, 23]. This has also been shown for the expression of prolactin in pituitary tissue [34]. This pulsatile gene expression averages out over time across the cells in the tissue, such that a reproducible expression is measured from the whole tissue. One could therefore speculate that OO patterns are somewhat linked to such pulsatile effects. For example, if cells that are on during a pulse fail to shut off, one would get on average a higher spontaneous expression across the tissue. Such an effect should become visible in single-cell data. For genes that show an OO in an organ from which the cells are derived, one would expect that a subset of the cells shows much higher expression than the cells from controls where no OO is observed. Alternatively, all or most cells show an elevated expression. This can be measured as spread of variance, whereby the variance should be high when only few cells express very highly. In our analysis, we measure indeed a higher variance for the OO genes, but the difference to the control genes is not very large. For a subset of comparisons, the variances for outlier expression are even lower than for control gene expression. Hence, while pulsatile gene expression may contribute to outlier expression in some cases, it appears that usually all cells in a given cell type can become subject to outlier expression in the respective individuals.

### Edge of chaos expression?

The modeling of gene regulatory networks has revealed that complex interactions within these networks can lead to chaotic dynamics. Gene networks are often highly nonlinear and sensitive to initial conditions, two hallmarks of chaotic systems. It has long been hypothesized that living cells are at the critical boundary between an organized and a disorganized state [35] and gene expression analysis experiments in cell cultures have supported this [36].

In fact, it has been suggested that the presence of chaos in gene regulatory networks may offer some advantages, such as enhancing the flexibility and adaptability of cellular systems in complex and changing environments. Chaotic systems can show some robustness to perturbations at a broader scale, although with unpredictability at a finer scale [37]. This has led to suggestions that gene regulatory networks evolve towards a state at the “edge of chaos” to confer robustness against environmental perturbations [38, 39]. Most interestingly, while the whole gene regulatory network could show stability, there could be sub-motives that show chaotic behavior [40]. Hence, it seems possible that the outlier gene expression patterns are a signature of an edge of chaos effect in the sense that small perturbations trigger them in some individuals, while the overall network controls them. This would predict that such over-expressions are transient, putting even more emphasis on the need to generate longitudinal data of gene expression for individuals at a population scale.

If the outlier expression is an effect of chaotic behavior in the regulatory network, then it should not only affect the extreme over-expression (OO), but also extreme under-expression (UO). We have not included a systematic analysis of UO patterns here, since they need more refined cutoff considerations, due to being naturally bounded by zero expression. However, a preliminary analysis with a cutoff of (Q1–3 × IQR) shows similar patterns as for OO, i.e., most individuals harbor only few UO genes, but some individuals show many. With a few exceptions, there is no correlation between the numbers of OO and UO in a given sample. Hence, this overall pattern would be in line with the edge of chaos assumption, including independently both, upregulation and downregulation effects.

## Conclusions

The occurrence of outlier gene expression patterns in individuals is a recurrent phenomenon across species, tissues, and cells. Many of the outliers occur as singletons; others in co-regulated modules. There is a large overlap between mouse and human outlier genes. This may imply that most, if not all, genes can potentially show sporadic overexpression in some individuals. Most of these expression effects are not driven by genetic variants. Extreme expression values are a biological reality that could indicate that gene regulatory mechanisms operate at the edge of chaos, which shows up in individuals at some point in their lifetime.

There is clearly a need to generate more data to investigate the phenomenon of outlier gene expression, especially as it may be relevant for the health status of individuals. On the other hand, for standard transcriptome studies that are aiming to reveal average biological effects, it remains advisable to filter out the outlier values from the datasets. But it could be useful to not simply discard these data, with the option to eventually revisit them for testing hypotheses on explaining the phenomenon of outlier expression.

## Methods

### Mouse data

Mouse data includes bulk RNA-Seq from wild mouse populations, an inbred mouse strain, and wild mouse families. Many of these data were part of a parallel study on sex-biased gene expression [11]. In this study we found that while outlier expression can affect the statistical analysis of sex-biased gene expression, it shows no specific sex-related patterns. For the present study we have therefore used both pure sex, as well as mixed-sex samples.

For the wild mouse populations, RNA-Seq data for the five different organs (brain, heart, kidney, liver, and mammary gland) were obtained together with the project described in [11]. In short, the data were collected from individuals kept under outbreeding conditions [41] from two *Mus musculus* subspecies, *M. m. domesticus* from France (DOM) and *M. m. musculus* (MUS) as well as two sister species, *M. spretus* (SPR) and *M. spicilegus* (SPI). For the DOM samples, we focused on 48 females (Additional file 1: Table S1A) which were collected with information for their estrous cycle. However, in an independent analysis, we did not find a systematic influence of the estrous cycle stage on the transcriptome patterns for the non-gonadal organs used in the current study (Xie et al. in preparation), i.e., we do not use estrous cycle stage as a factor for the further analysis. For the other outbred animals, we used both males and females; a total of 19 individuals were used for MUS and SPR, and 20 for SPI (Additional file 1: Table S1B). Note that the five organs used were always from the same individuals. In total, data from 63 individuals are also used in the project described in [11], and the rest from 43 individuals are only used in the present project, but all the data were obtained in parallel at the same time by the same people and with the same procedure.

For the comparison with the inbred strain C57BL/6, we sequenced the brain RNA from 24 animals from the corresponding inbred stock (Additional file 1: Table S1E).

For the wild mouse families used in the outlier inheritance analysis, five three-generation families of an outbred stock of *M. m. domesticus* from Germany were set up, and each family has ten individuals. The breeding schemes are shown on the top of Fig. 3 and in Additional file 11: Table S11A. Brain, kidney, and Liver RNA-Seq were generated for each individual. The steps for assigning the inheritance state for each gene are described in the readme tab of Additional file 11: Table S11.

### Human data

Human data includes bulk RNA-Seq from the GTEx project [42, 43], single-nucleus RNA-Seq (snRNA-Seq) from the SEA-AD project [24], and bulk multiomics data from the “Rush Alzheimer’s Disease Study” project (“https://www.encodeproject.org/brain-matrix/?type=Experiment&status=released&internal_tags=RushAD”).

The human bulk RNA-Seq data were retrieved from the GTEx project as TPM files for each organ. To make them best comparable with the mouse data, we chose three organs that were also analyzed for the mouse, brain (cortex), heart (left ventricle), and Liver. We retrieved 51 individuals from which data were available for each of these organs. In addition, we also used pituitary data from 40 of the 51 individuals (Additional file 1: Table S1C).

The human snRNA-Seq data were retrieved from the SEA-AD project as “h5ad” format files, including the normalized read counts presented as a log transformation of pseudocounts per 10,000 reads, ln(CPTT + 1), which were downloaded from CZ CELLxGENE [44, 45]. Cells in two brain regions were included: middle temporal gyrus (MTG) and dorsolateral prefrontal cortex (DLPFC). The data from the individuals with race as “white”, and sequenced by the “10 × 3′ v3” assay was used for further selection. We selected 53 individuals and 24 cell types (“subclass”, 15 for DLPFC and nine for MTG), and each of the 24 cell types has at least 100 cells for each of the 53 individuals. The full List of cell types and individuals included is provided in Additional file 1: Table S1F. We used CPTT as the expression level of a gene in a cell, and mean(CPTT) as the expression level of a gene in a cell type of a brain region from an individual.

The bulk multiomics data of dorsolateral prefrontal cortex samples from the Rush AD project were downloaded from ENCODE [26]. We chose 45 individuals with RNA-Seq, DNase-Seq, CTCF ChIP-Seq, H3K4me3 ChIP-Seq, H3K27ac ChIP-Seq, and H3K27me3 ChIP-Seq data from the same samples. For RNA-Seq data, TPM values of all human genes were extracted from the “gene quantifications” files. For the remaining data, fold enrichment values of all detected peaks were extracted from the “peaks” files. All data are included in Additional file 15: Table S14.

### *Drosophila* data

*Drosophila* data includes bulk RNA-Seq from *D. melanogaster* [13] and *D. simulans* [14]. The *D. melanogaster* data includes the RNA-Seq of heads and bodies from 27 females, and the CPM files were provided by the authors. The *D. simulans* data includes the RNA-Seq of the whole flies from four populations, with 19 to 22 males from each population, and the CPM files were provided in the supporting information of the paper (Additional file 1: Table S1D).

### RNA sequencing and data analysis

The mouse organs were carefully dissected and immediately frozen in liquid nitrogen. Total RNAs were purified using QIAGEN kits, QIAzol (Catalog no. 79306) and RNeasy 96 Universal Tissue Kit (Catalog no. 74881), and prepared using Illumina TruSeq Stranded mRNA Kit, and sequenced using Illumina NovaSeq S4 (2 × 150 bp) in Kiel Sequencing Center (for all samples except for the five technical replicates of the pedigree samples) or Illumina NextSeq 500 (2 × 150 bp) on the MPI Plön sequencing machines (for the five technical replicates of the pedigree samples). All procedures were performed in a standardized and parallel way to reduce experimental variance.

Raw sequencing reads were trimmed using Trimmomatic (0.38) [46] with suggested parameter settings, except we did not remove bases from the beginning of reads. Only paired-end reads left were used for following analyses. The trimmed reads were mapped to mouse genome GRCm39 [47, 48] using STAR (2.7.9a) [49]. Based on the communication with the authors of STAR, the parameters were adjusted to account for the sequence divergence of the mouse taxa and the reference genome: “–outFilterMismatchNmax 30 –scoreDelOpen −1 –scoreDelBase −1 –scoreInsOpen −1 –scoreInsBase −1 –seedSearchStartLmax 25 –winAnchorMultimapNmax 100”. Duplicate reads were removed with PICARD (2.9.0) (http://broadinstitute.github.io/picard). Note that duplicate removal is usually not performed for RNA-Seq analysis, but considering that we were looking for extreme gene expression, we wanted to make sure the signals found were not due to duplicates. Fragments mapped to the genes annotated by Ensembl (Version 104) were counted using featureCounts (2.0.3) [50].

### Assignment of extreme gene expression

For a specific gene in a specific sample population, we required that at least one TPM value (CPM value for *Drosophila*, and mean(CPTT) value for human snRNA-Seq) is larger than 5. If a TPM value is larger than Q3 + 5 × IQR and larger than 5, it is assigned to be an extreme over outlier (OO), and if a TPM value is smaller than Q1–5 × IQR, it is assigned to be an extreme under outlier (UO).

For sample populations containing two sexes, such as the sample populations of MUS, SPR, SPI, C57BL/6, mouse pedigree, and human, we additionally performed Fisher’s exact test of independence between outlier assignment and sex for each gene. When the *P*-value is smaller than the marginal significance cutoff 0.1, we removed the gene from the extreme outlier gene set, because its expression might just be sex-biased instead of extreme in a few samples. For human sample populations, unlike mouse and fruit fly sample populations which are well controlled during animal experiments in the lab, besides sex, age and death type also introduce significant heterogeneity. Thus, we also performed Fisher’s exact test of independence between outlier assignment and age/death type separately for each gene, and removed the gene from the extreme outlier gene set if any *P*-value is smaller than 0.1.

Individual checking of read coverage in annotated non-coding genes showed a number of cases where outlier read mapping covered only a small portion of the genes. While this is in itself an interesting observation, we did not strive to further explore it here. Hence, all the analysis was restricted to protein-coding genes only, which represent roughly 2/3 of the outlier calls among all transcripts.

If a gene and its paralog have high sequence similarity, read alignment Might be problematic. Thus, we filtered out genes having at least one paralog with identity larger than 90% calculated by Ensembl Compara [48]. This cutoff might be too stringent; alignment tools, such as STAR, can also perform well on genes with high sequence similarities. But we wanted to make sure the signals found were not due to this problem.

### Subsampling analysis

To evaluate the effects on the cutoff (fold of IQR) and sample size for outlier calling, we used subsampling analysis from the mouse dataset of the 48 DOM individuals. We first excluded one sample from each of the five organs, which contains a very large number of outlier genes and could lead to an uninformative large variance during subsampling. The samples excluded are: H35180 (individual 38) for brain, H35232 (individual 18) for heart, H35342 (individual 35) for kidney, H35302 (individual 31) for liver, and H35425 (individual 30) for mammary gland. For cutoff evaluation, we tested fold of IQR from 3 to 10. For each organ and each fold of IQR, we did 1000 subsampling runs. In each run, we randomly chose 24 samples from the 47 samples in total, and performed the assignment analysis of extreme gene expression as described above. For sample size evaluation, we used 5 as the fold of IQR and tested the sample size N as 8, 16, 24, 32, and 40. For each organ and each sample size, we also did 1000 subsampling runs. In each run, we randomly chose N samples from the 47 samples in total, and performed the assignment analysis of extreme gene expression as described above.

### Human snRNA-Seq analysis

For the assignment of extreme gene expression in each cell type of the human snRNA-Seq data, we used the mean of the counts per 10,000 reads, mean(CPTT), of all the cells belonging to the cell type in an individual as the expression level of a gene, and then performed the same analysis described above.

For the analysis of the expression variance of OOs, we used the counts per 10,000 reads, CPTT, as the expression level of a gene in a cell. For each of the 307 OOs belonging to the 145 genes, we calculated the coefficient of variation (CV) of the gene among the cells of the cell type and the individual with which the OO exists. In addition, we also chose a control gene for the OO in the same cell type and the same individual. The rule of the choice is that the mean(CPTT) of the control gene of the individual is within the Q1 to Q3 range across individuals of the cell type, and the mean(CPTT) of the control gene is close to the mean(CPTT) of the OO. Then, we also calculated the CV of the control gene in the same way.

### Rush AD data analysis

For the assignment of extreme gene expression in the RNA-Seq data of 45 individuals, the same analysis described above was performed. Then, for the detected outlier genes, fold enrichment values of the peaks detected in DNase-Seq, CTCF ChIP-Seq, H3K4me3 ChIP-Seq, H3K27ac ChIP-Seq, and H3K27me3 ChIP-Seq were analyzed in the regions around their transcriptional start sites (TSSs), i.e., TSS ± 2000 bp, which were called “promoter” regions here. For H3K27me3 ChIP-Seq, fold enrichment values were also analyzed in the gene body regions (“H3K27me3_gb”). If a region contains no peaks, its fold enrichment value is assigned as zero; and if a region contains one peak, its fold enrichment value is assigned as the value of the peak; and if a region contains multiple peaks, its fold enrichment value is assigned as the sum of the fold enrichment values of the peaks.

### Human protein data analysis

Blood samples from the NHS and NHSII were analyzed for Prolactin using Microparticle enzyme immunoassays in 11 batches using the ARCHITECT chemiluminescence immunoassay system (Abbott Diagnostics), and an additional three batches were assayed using the IMx System (Abbott Laboratory) [30]. GH was assayed by ELISA using reagents from Diagnostic Systems Laboratory [31]. 

## Supplementary Information

Additional file 1: Table S1. Sample lists for all data used in the study (Excel file with 7 tabs). Table S1A: mouse DOM population samples. Table S1B: mouse MUS, SPR and SPI population samples. Table S1C: human GTEX samples. Table S1D: *Drosophila melanogaster* and *Drosophila simulans* samples. Table S1E: mouse inbred strain C57BL/6 samples. Table S1F: human samples from sn-study. Table S1G: human samples from Rush AD study.

Additional file 2: Table S2. Gene lists with transcriptome data (TPM) for five organs of the mouse DOM populations (Excel file with ten tabs). Organ names are provided in the Tab titles. For each organ, "all_TPM"includes data for all genes above the minimal expression cutoff value, "OO"is the corresponding sub list for all genes with at least one over-outlier expression.

Additional file 3: Table S3. Gene lists with transcriptome data (TPM) for five organs of the mouse MUS populations (Excel file with ten tabs). Organ names are provided in the Tab titles. For each organ, "all_TPM"includes data for all genes above the minimal expression cutoff value, "OO"is the corresponding sub list for all genes with at least one over-outlier expression.

Additional file 4: Table S4. Gene lists with transcriptome data (TPM) for five organs of the mouse SPR populations (Excel file with ten tabs). Organ names are provided in the Tab titles. For each organ, "all_TPM"includes data for all genes above the minimal expression cutoff value, "OO"is the corresponding sub list for all genes with at least one over-outlier expression

Additional file 5: Table S5. Gene lists with transcriptome data (TPM) for five organs of the mouse SPI populations (Excel file with ten tabs). Organ names are provided in the Tab titles. For each organ, "all_TPM"includes data for all genes above the minimal expression cutoff value, "OO"is the corresponding sub list for all genes with at least one over-outlier expression.

Additional file 6: Table S6. Gene lists with transcriptome data (TPM) for brain of the mouse inbred strain C57BL/6 (Excel file with two tabs). "BL6_brain_TPM_all"includes data for all genes above the minimal expression cutoff value, "BL6_brain_OO"is the corresponding sub list for all genes with at least one over-outlier expression

Additional file 7: Table S7. Gene lists with transcriptome data (TPM) for four organs of the human GTEx data (Excel file with eight tabs). Organ names are provided in the Tab titles. For each organ, "all_TPM"includes data for all genes above the minimal expression cutoff value, "OO"is the corresponding sub list for all genes with at least one over-outlier expression.

Additional file 8: Table S8. Gene lists with transcriptome data (CPM) for *Drosophila* data (Excel file with twelve tabs). For *Drosophila melanogaster* (Dmel) there are two parts (head and body), for *Drosophila simulans* (Dsim) there are four populations, as indicated in the tabs. In each case, "all" includes data for all genes above the minimal expression cutoff value, "OO" is the corresponding sub list for all genes with at least one over-outlier expression

Additional file 9: Table S9. Analysis details for the distribution of outlier genes among tissues and species (Excel file with ten tabs). Table S9A: lists of outlier genes occurring in more than one mouse organ in DOM. Table S9B: OO patterns of mouse genes that are expressed in more than one tissue. Table S9C: lists of brain outlier genes occurring in more than one mouse taxon. Table S9D: lists of heart outlier genes occurring in more than one mouse taxon. Table S9E: lists of kidney outlier genes occurring in more than one mouse taxon. Table S9F: lists of liver outlier genes occurring in more than one mouse taxon. Table S9G: lists of mammary outlier genes occurring in more than one mouse taxon. Table S9H: lists of outlier genes occurring in more than one human organ. Table S9I: OO patterns of human genes that are expressed in more than one tissue. Table S9J: lists of mouse and human orthologous genes that show OO patterns in the data.

Additional file 10: Table S10. List of outlier genes with replication data from two sequencing experiments (Excel table).

Additional file 11: Table S11. Pedigree and data for the mouse family analysis (Excel file with five tabs). Table S11A: pedigree scheme for the five families. Table S11B: data and analysis for brain. Table S11C: data and analysis for kidney. Table S11D: data and analysis for liver. Table S11E: subset of data and analysis for genes that follow Mendelian segregation ratios

Additional file 12: Table S12. Data for outlier genes occurring in modules in a semi-graphic depiction (Excel file with three tabs). Table S12A: Depiction of mouse outlier modules based on shared OO in at least three individuals for gene pairs and larger groups of genes. Table S12B: Depiction of human outlier modules based on shared OO in at least three individuals for gene pairs and larger groups of genes. Tale S12C: Depiction of *Drosophila* outlier modules based on shared OO in at least three individuals for gene pairs and larger groups of genes. 

Additional file 13: Table S13. Data and analysis for the data from the single nuclei sequencing experiments in human brain (Excel table with two tabs). Table S13A: list of individuals and cell types analyzed. Table S13B: variance comparisons for the OO genes.

Additional file 14: Figure S1. Depiction of chromatin marks around the *APOA1* gene in two human individuals

Additional file 15: Table S14. Data and analysis for the search for epigenetic signatures of outlier expression (Excel table). 

Additional file 16: Figure S2. Visualization of the variance data from Table S14

## Data Availability

Mouse data: The ENA BioProject accession numbers for the sequencing data reported in this study are: PRJEB50011 for the wild mouse populations [51], PRJEB75700 for the C57BL/6 inbred mouse strain [52], and PRJEB75139 for the wild mouse families [53]. Human data: Human data includes bulk RNA-Seq from the GTEx project [42, 43] https://www.gtexportal.org/home/, the snRNA-Seq data were retrieved from the SEA-AD project [24] (https://portal.brain-map.org/explore/seattle-alzheimers-disease), and bulk multiomics data from the ENCODE [26]"Rush Alzheimer´s Disease Study"project (“https://www.encodeproject.org/brain-matrix/?type=Experiment&status=released&internal_tags=RushAD”). *Drosophila* data: *Drosophila* data includes bulk RNA-Seq from *D. melanogaster* and *D. simulans* deposited under ENA BioProject accesion number PRJEB 37011 [54]. The code for our outlier detection is available at GitHub: https://github.com/cxiepku/outlier [55] under the MIT License and Zenodo [56] under the Creative Commons Attribution 4.0 International License.
