# Supplementary material for: Patterns of extreme outlier gene expression suggest an edge of chaos effect in transcriptomic networks
Source: Genome Biol. 2025 Sep 9;26:272. doi: 10.1186/s13059-025-03709-0 (PMC12418659; doi:10.1186/s13059-025-03709-0)

**Additional file 14: Figure S1**

*Depiction of chromatin marks around the APOA1 gene in two human individuals*


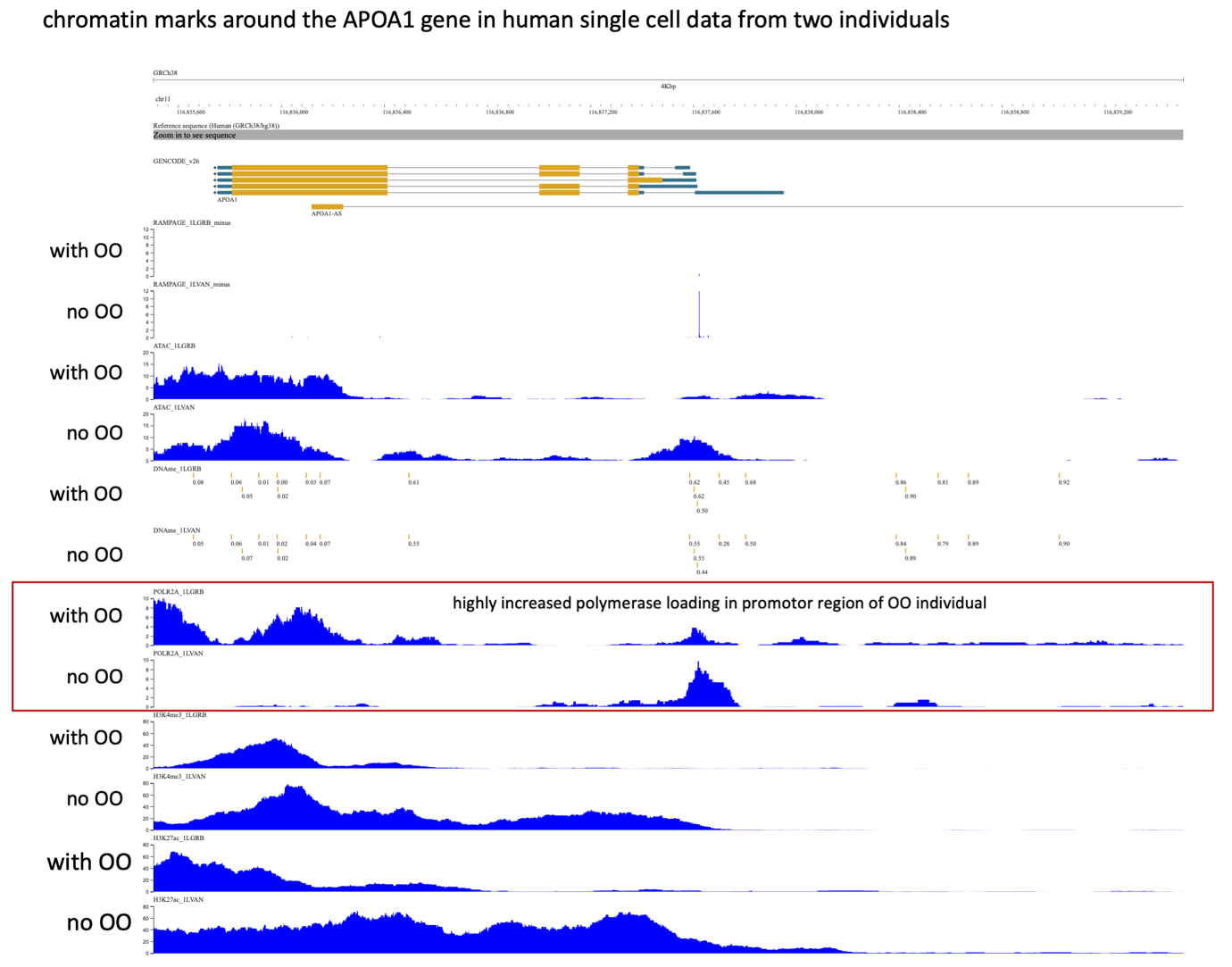

Supplement: Supplementary file 14 — Additional file 14: Figure S1. Depiction of chromatin marks around the APOA1 gene in two human individuals [file 13059_2025_3709_MOESM14_ESM.docx]
