# Supplementary material for: Patterns of extreme outlier gene expression suggest an edge of chaos effect in transcriptomic networks
Source: Genome Biol. 2025 Sep 9;26:272. doi: 10.1186/s13059-025-03709-0 (PMC12418659; doi:10.1186/s13059-025-03709-0)

**Additional file 16: Figure S2**

*Visualization of the variance data from Table S14*

Each row corresponds to the gene designated at the top of each graph, each column to the different epigenetic mark experiments. Dots represent the individuals with the RNA TPM values on the Y-axis and the different epigenetic mark measures on the X- axis. The red dots indicate the OO individuals.

Figure S2 - part1


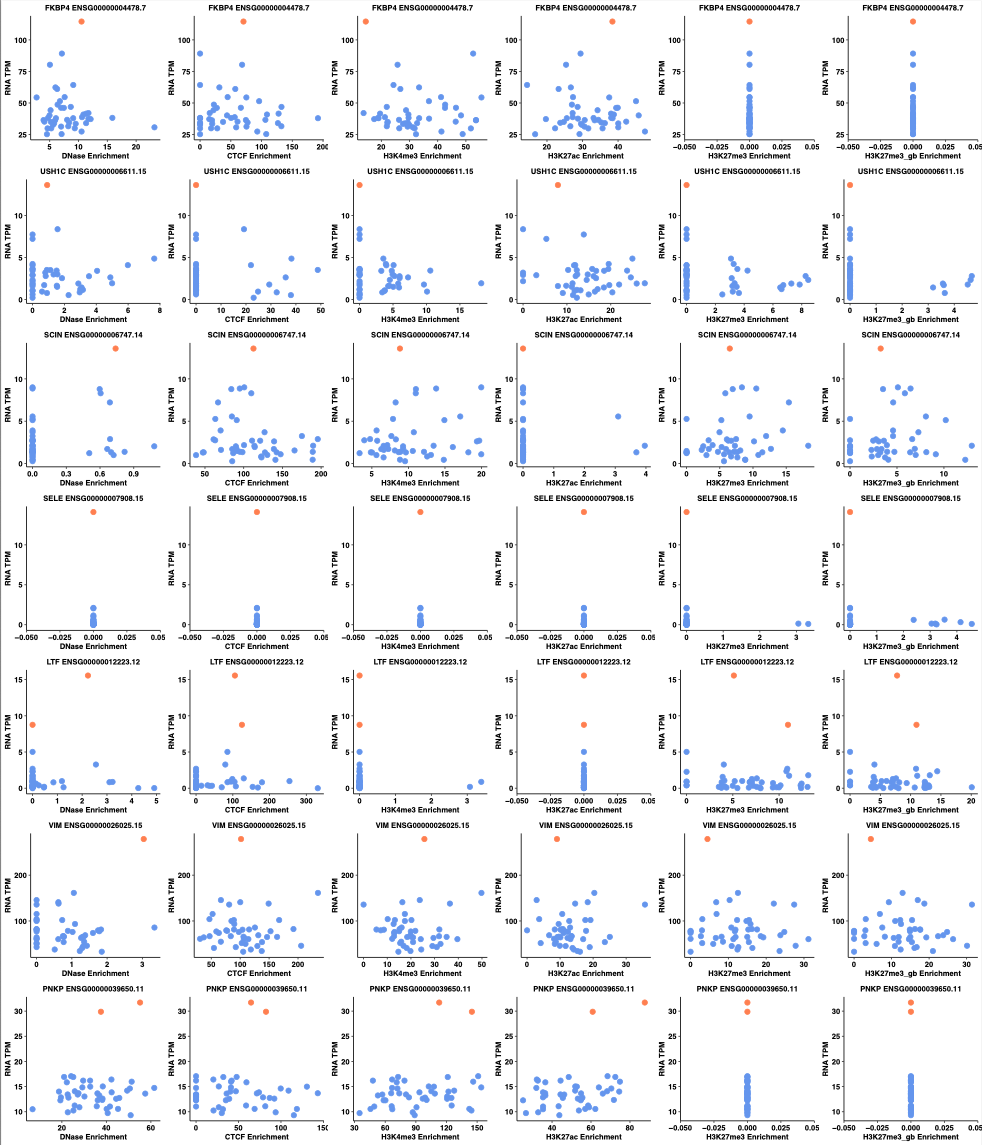


Figure S2 - part2


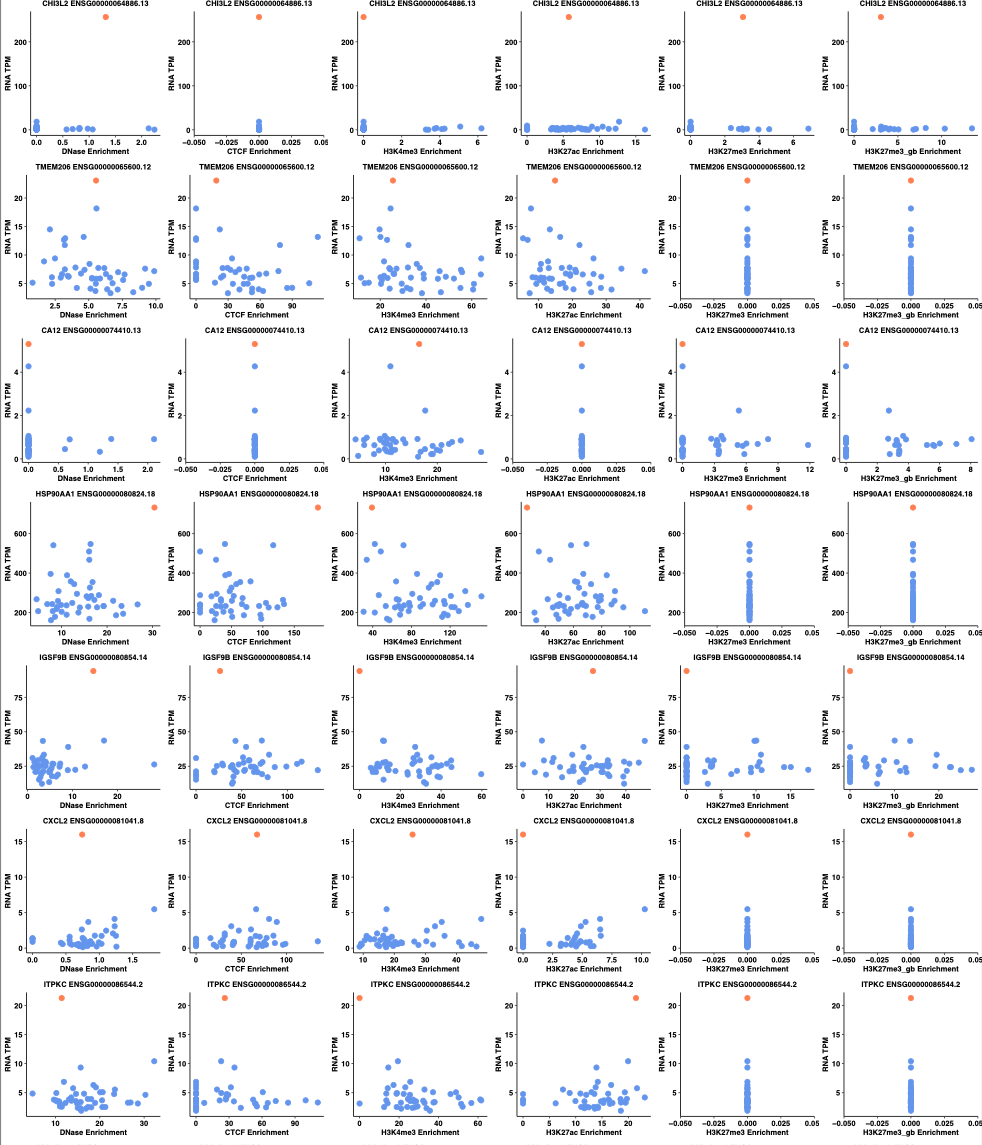


Figure S2 - part3


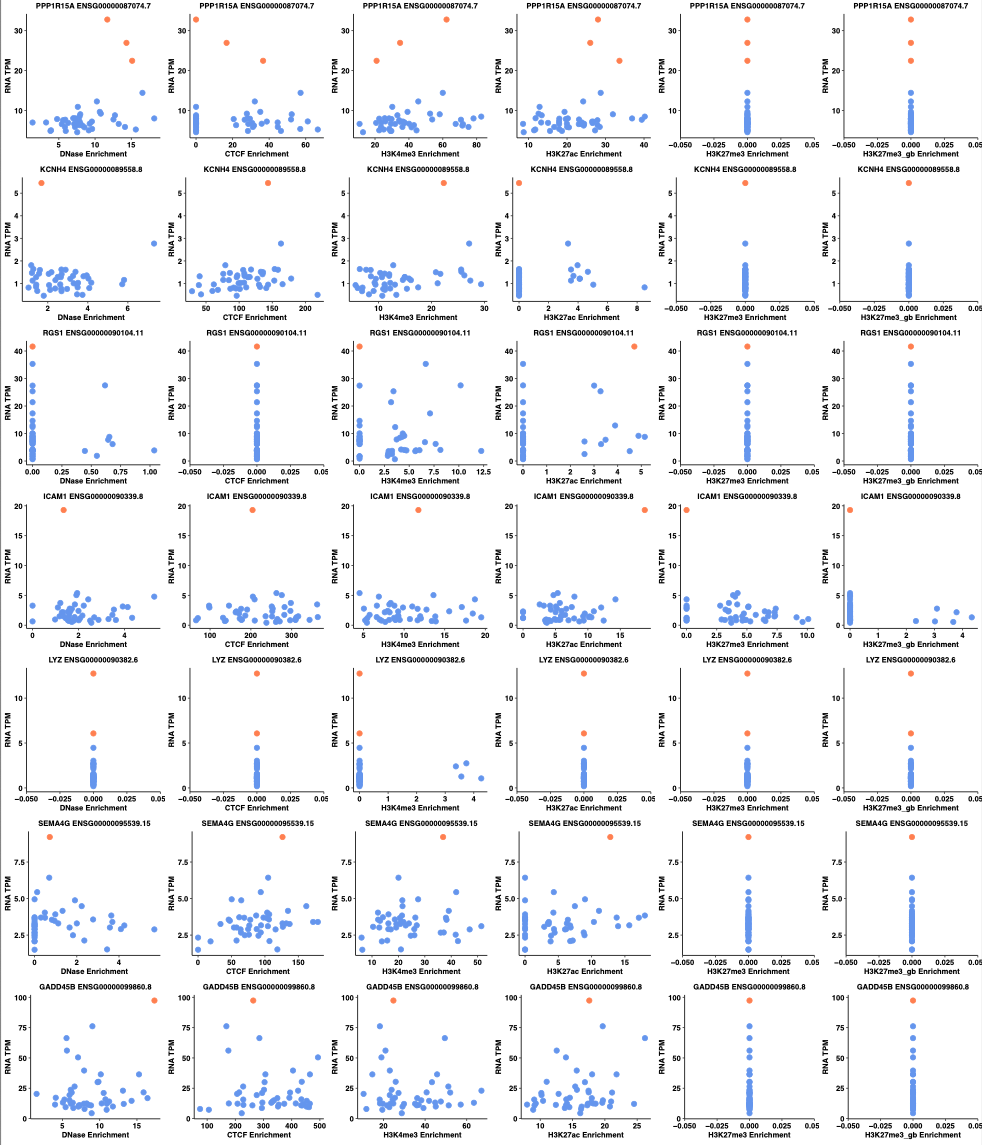


Figure S2 - part4


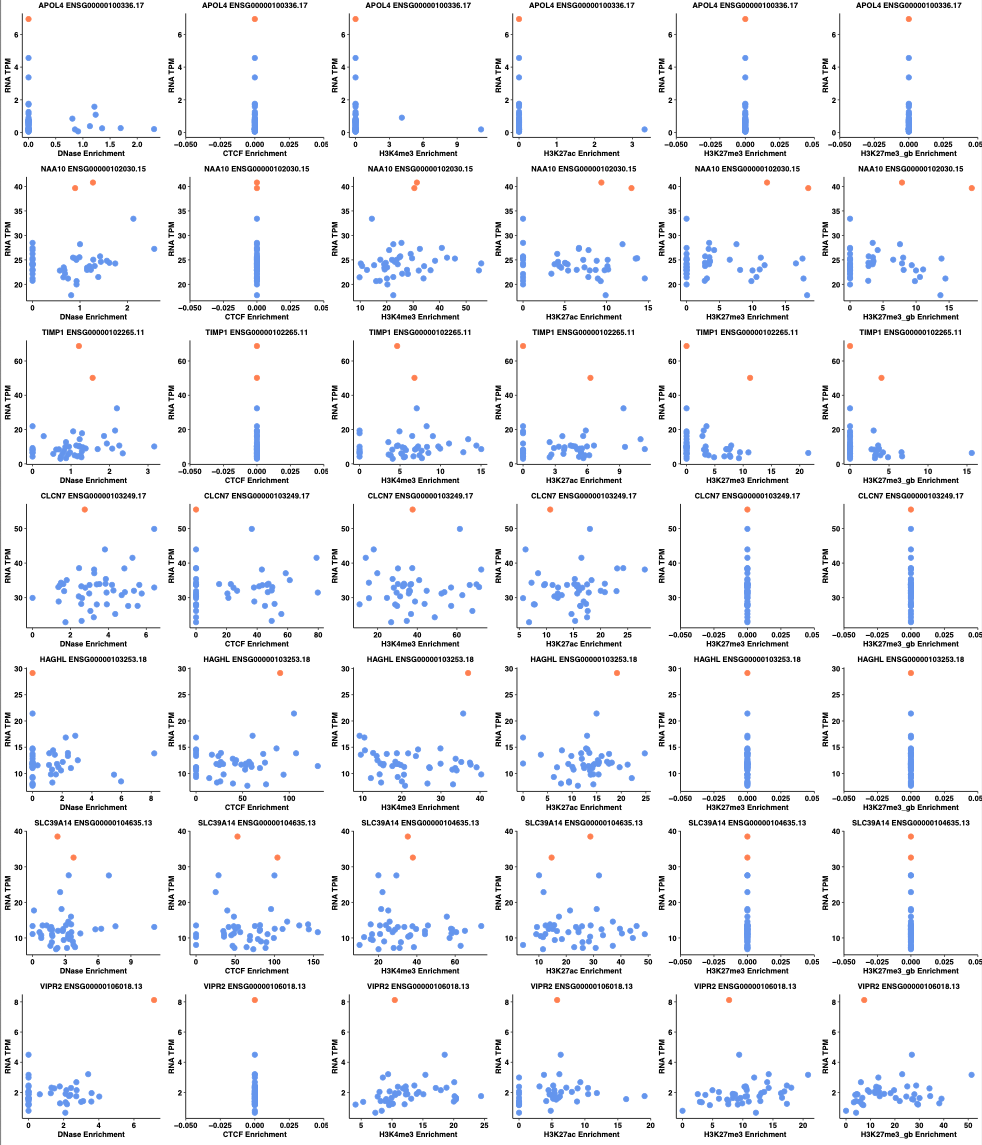


Figure S2 - part5


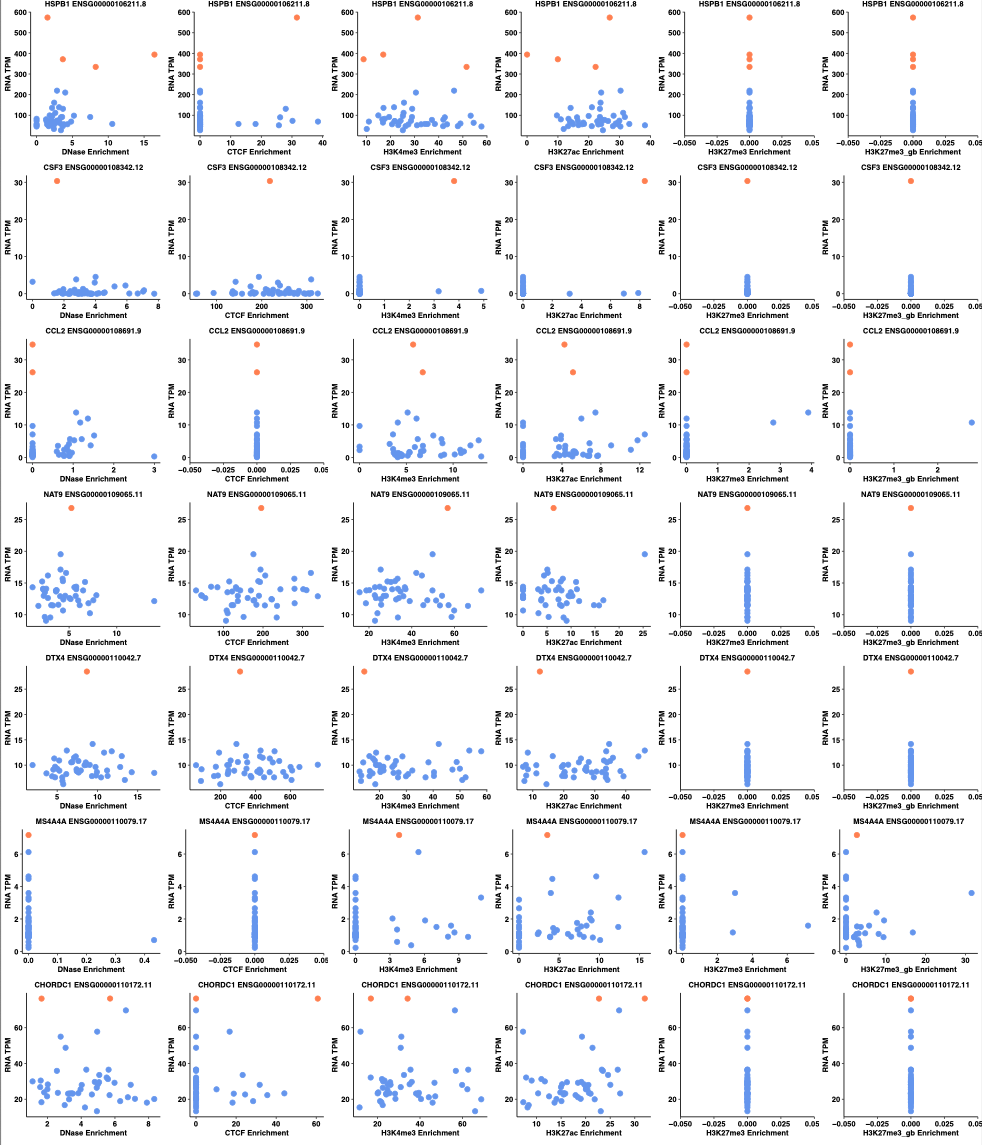


FigureS2 - part6


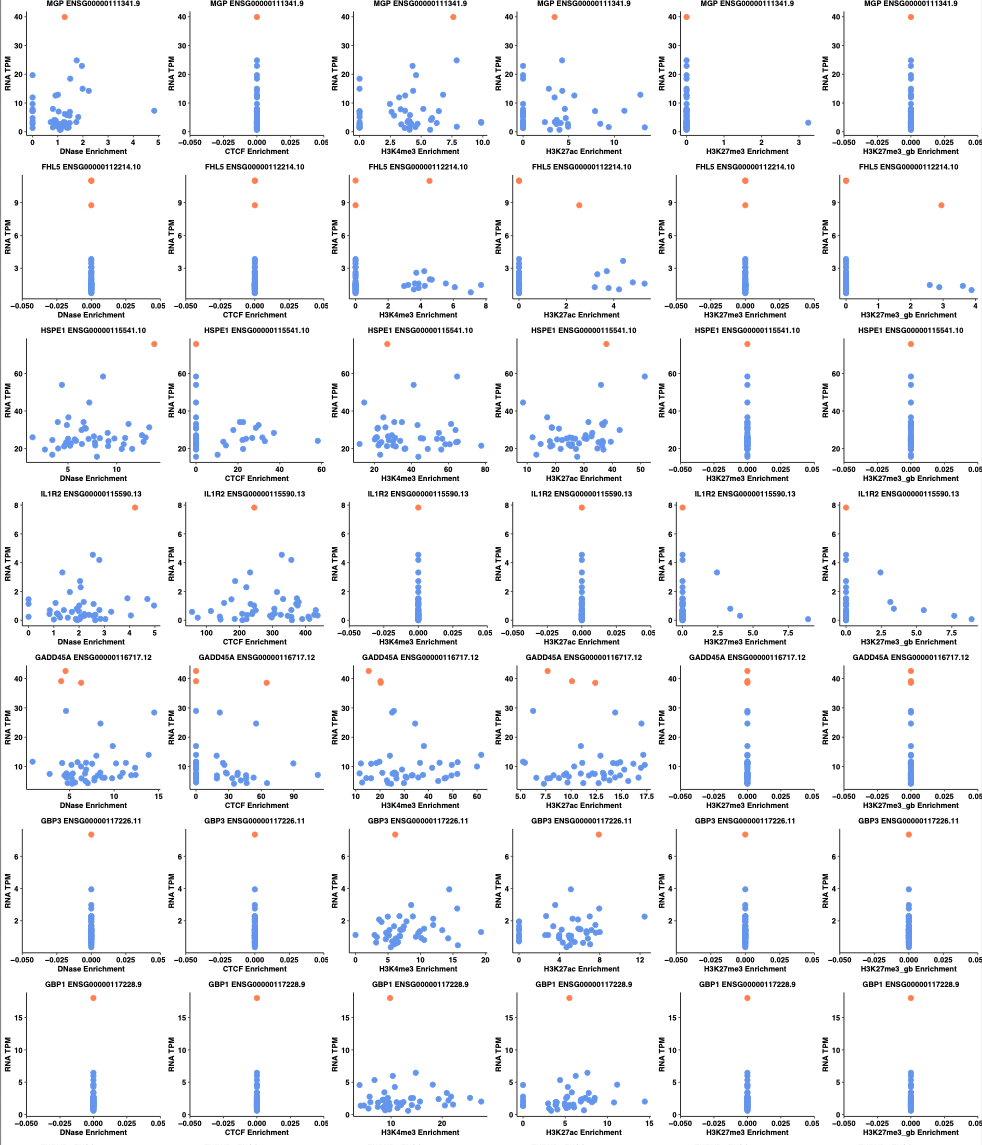


FigureS2 - part7


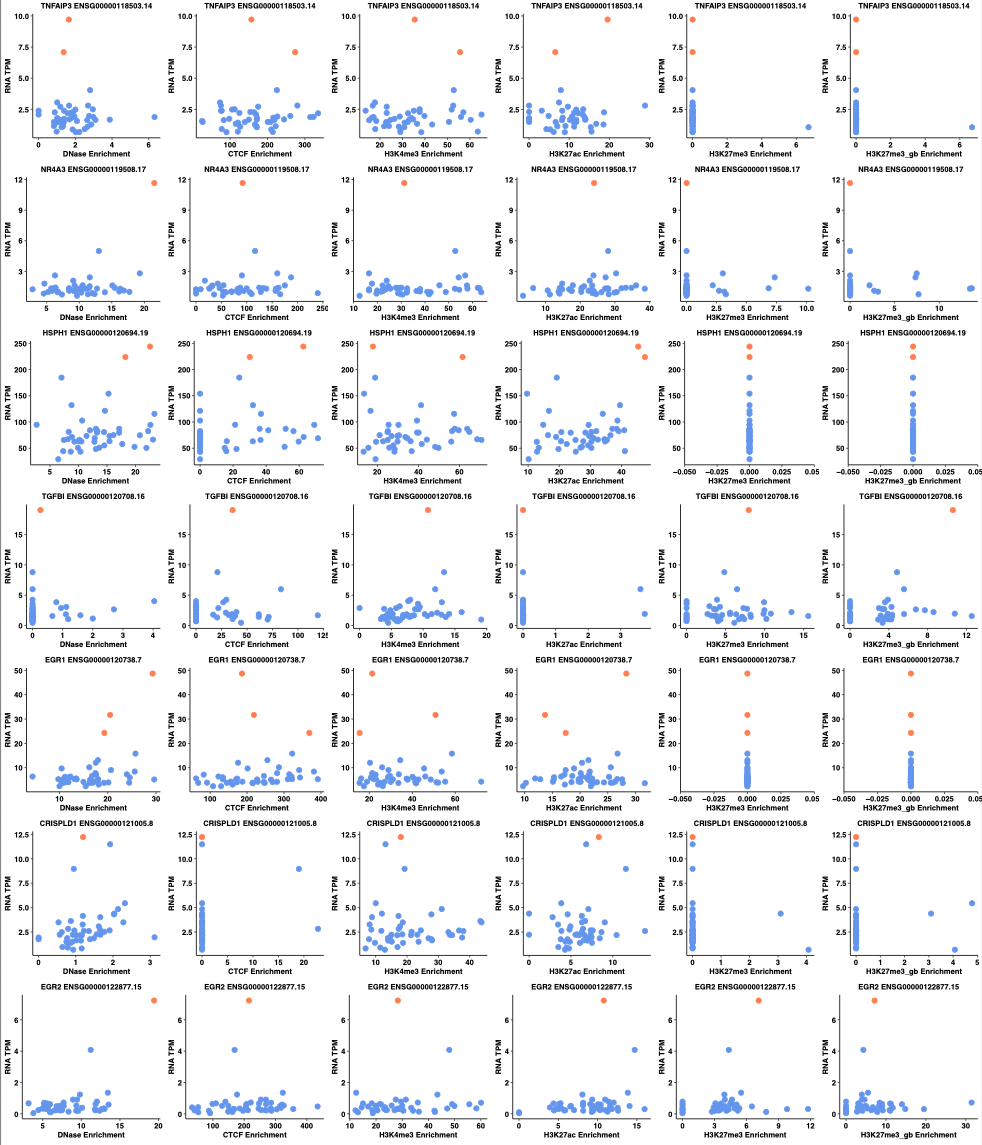


FigureS2 - part8


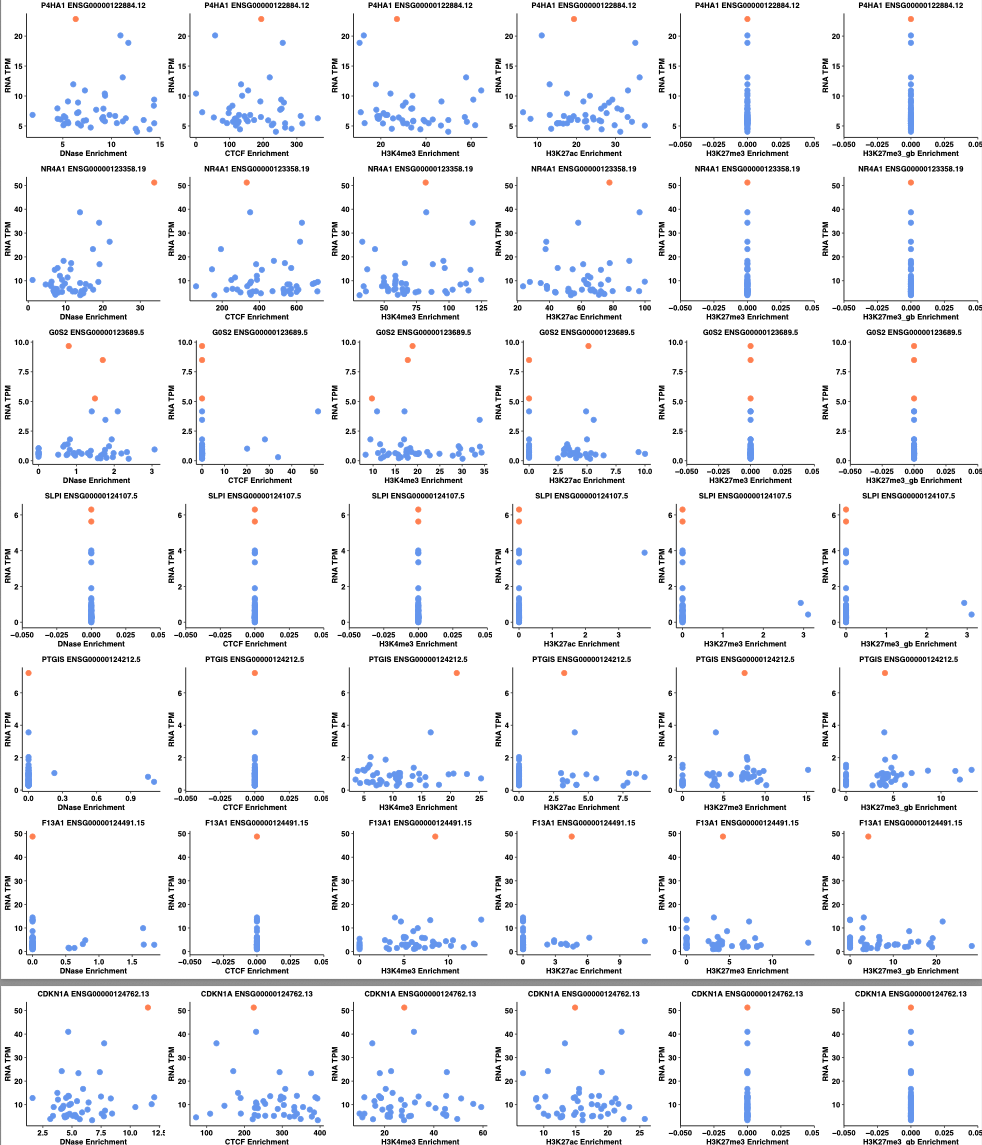


FigureS2 - part9


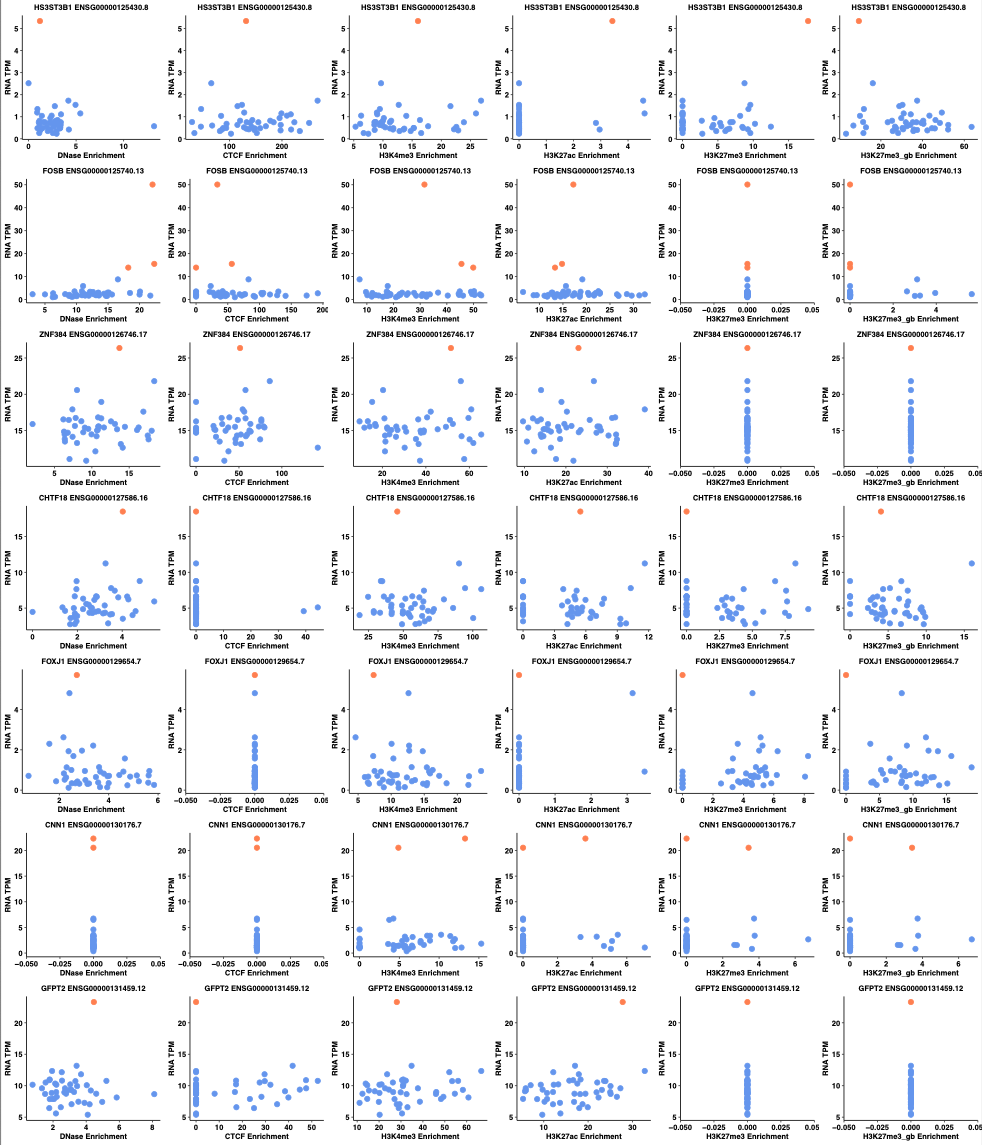


FigureS2 - part10


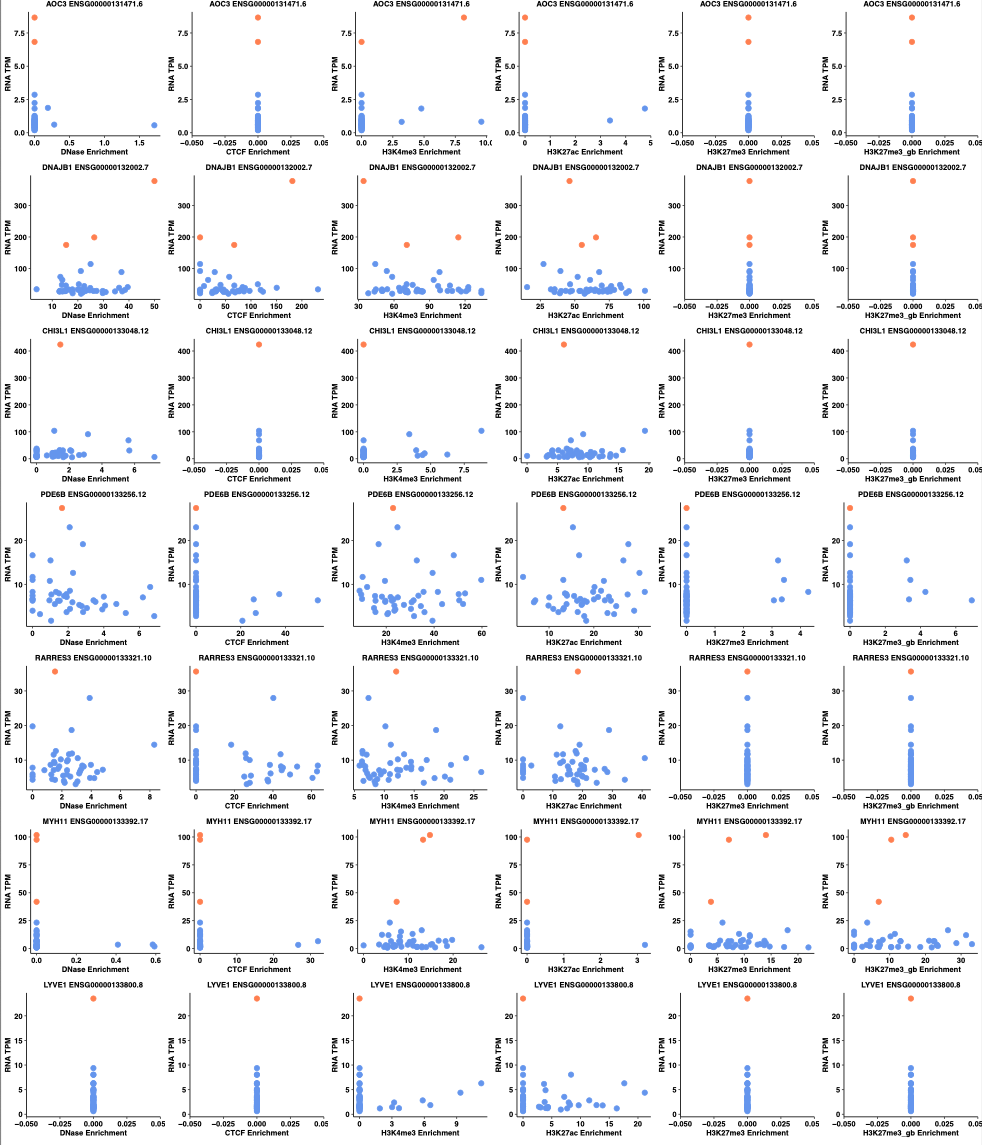


FigureS2 - part11


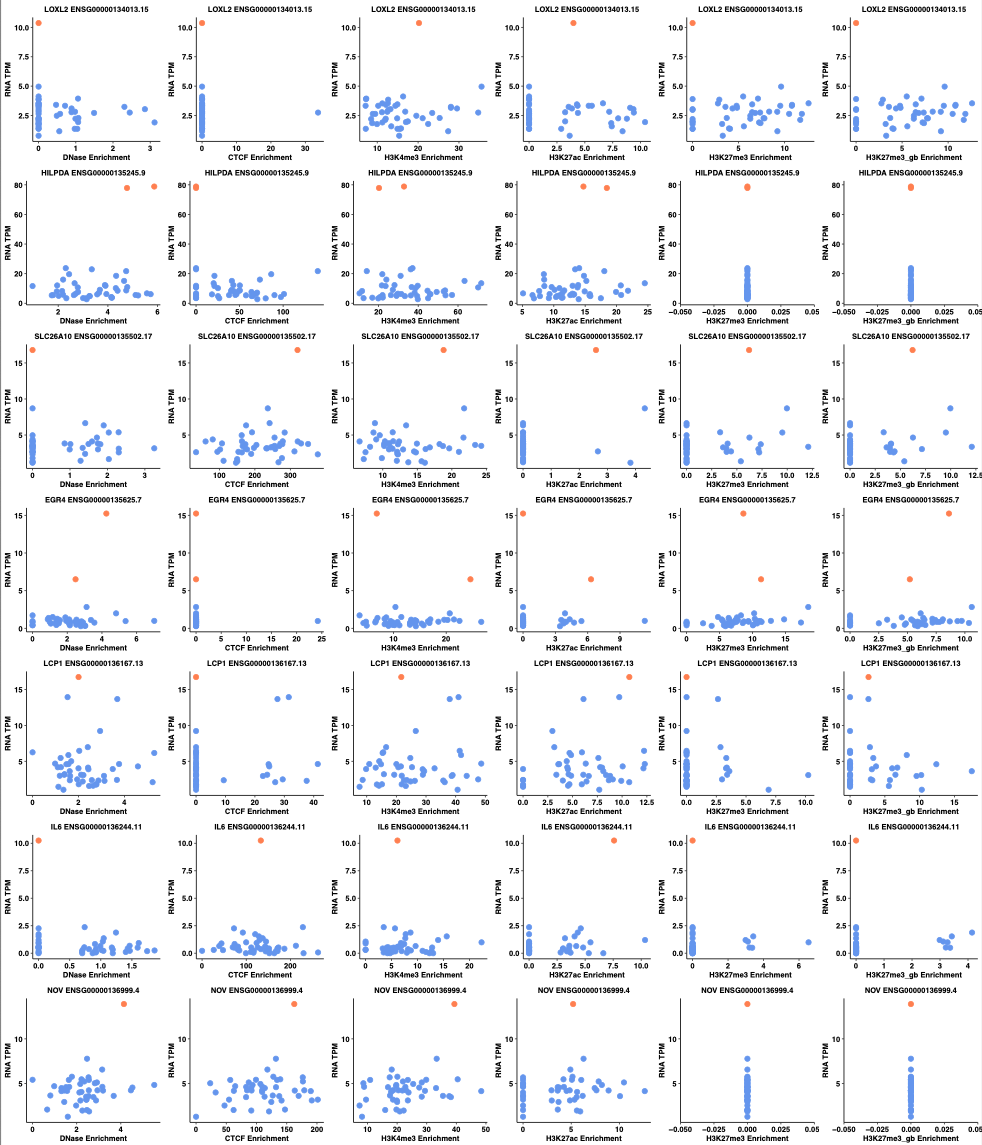


FigureS2 - part12


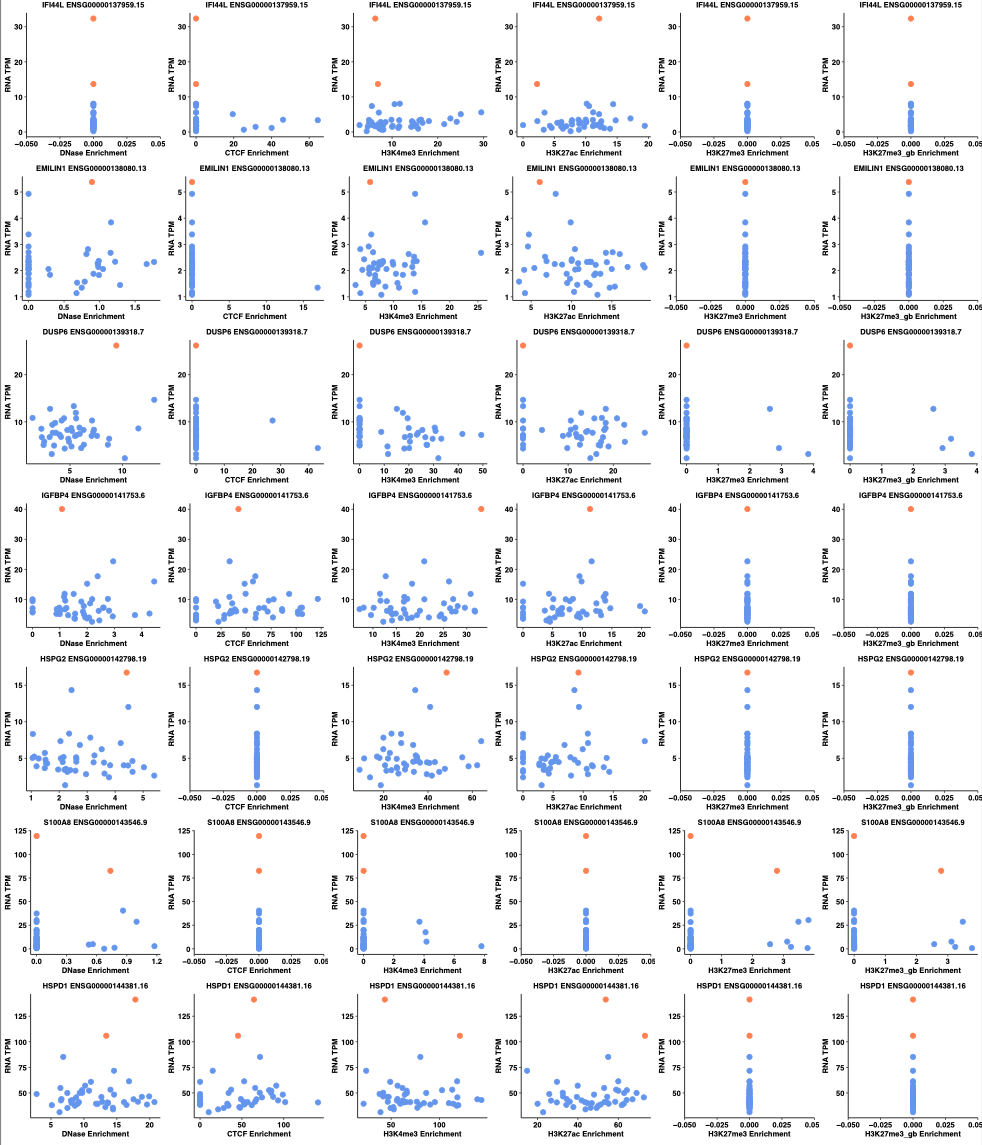


FigureS2 - part13


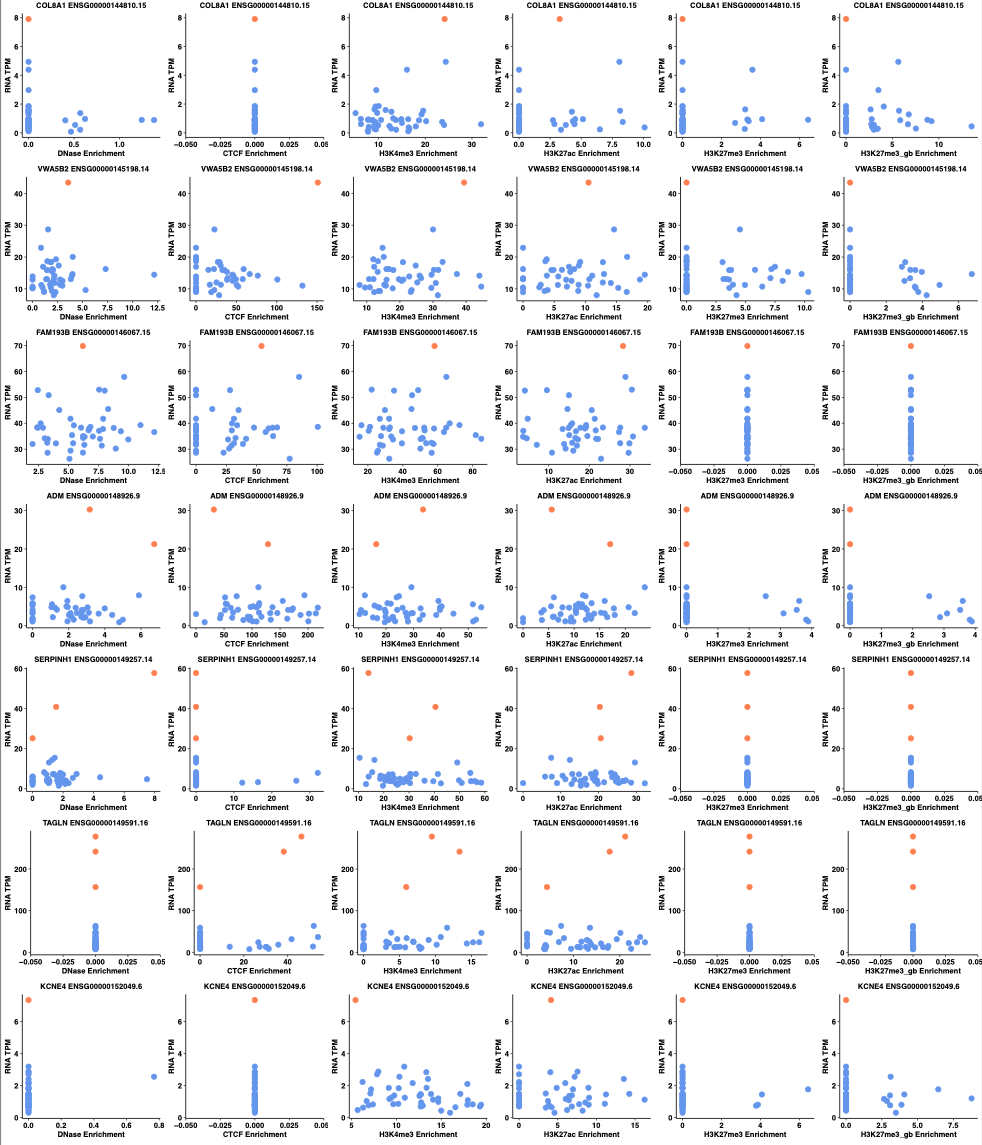


FigureS2 - part14


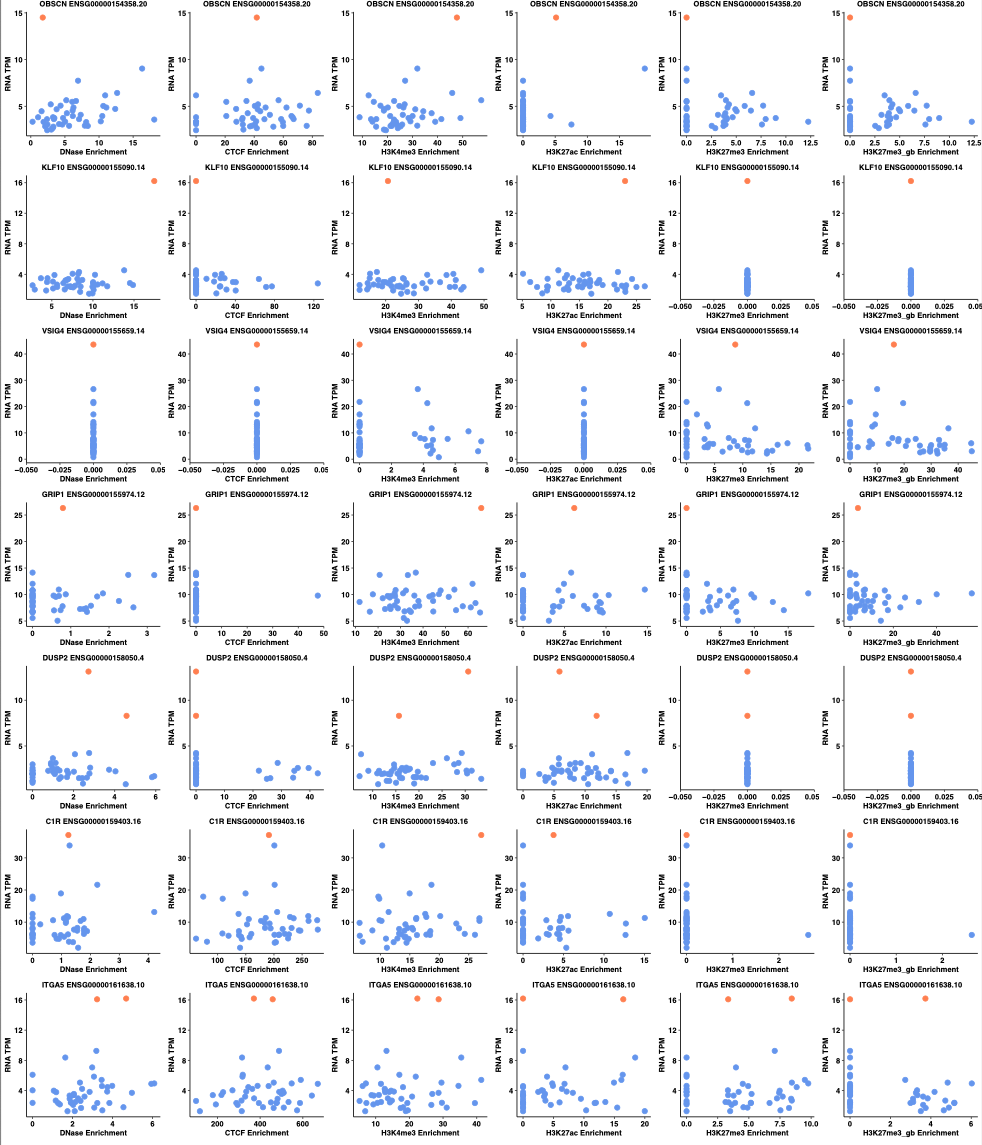


FigureS2 - part15


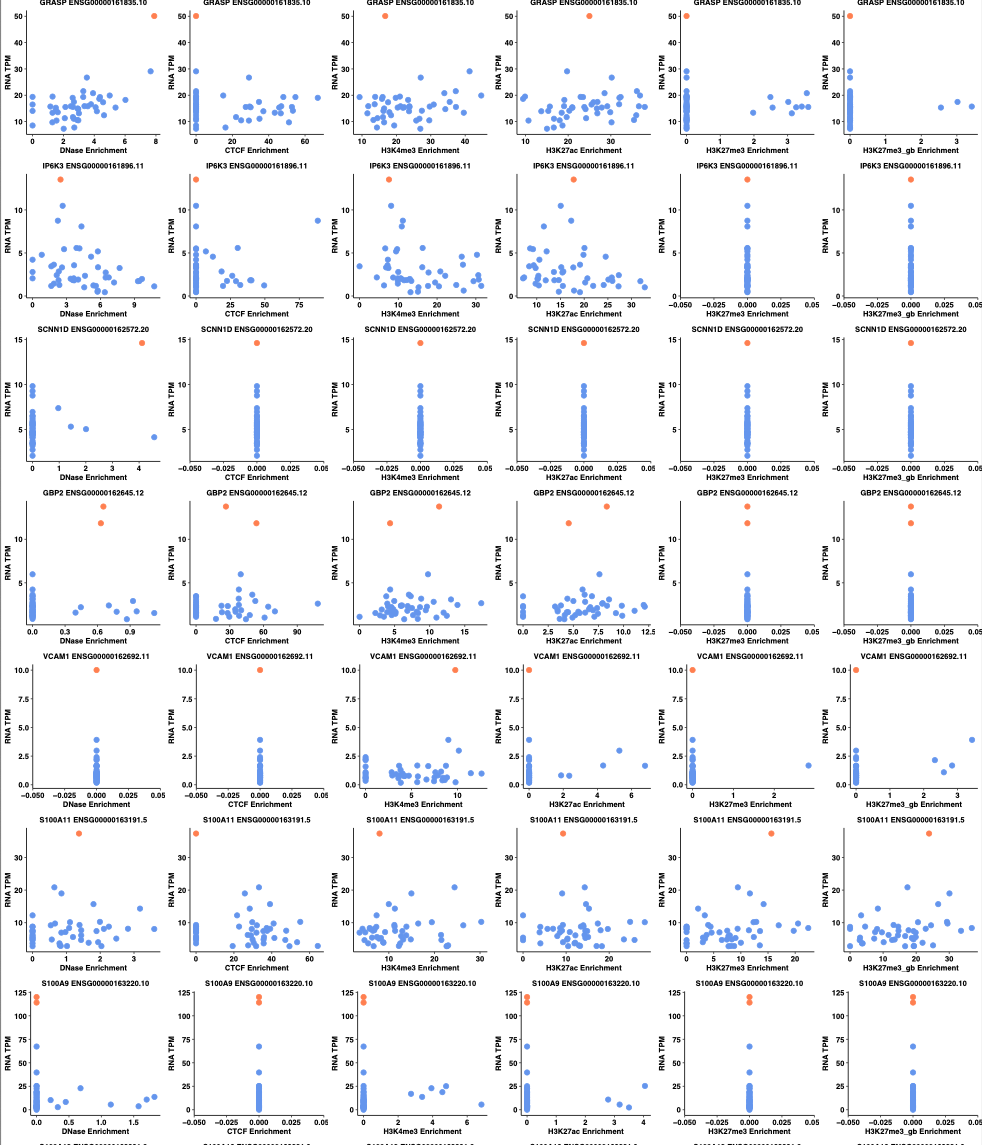


FigureS2 - part16


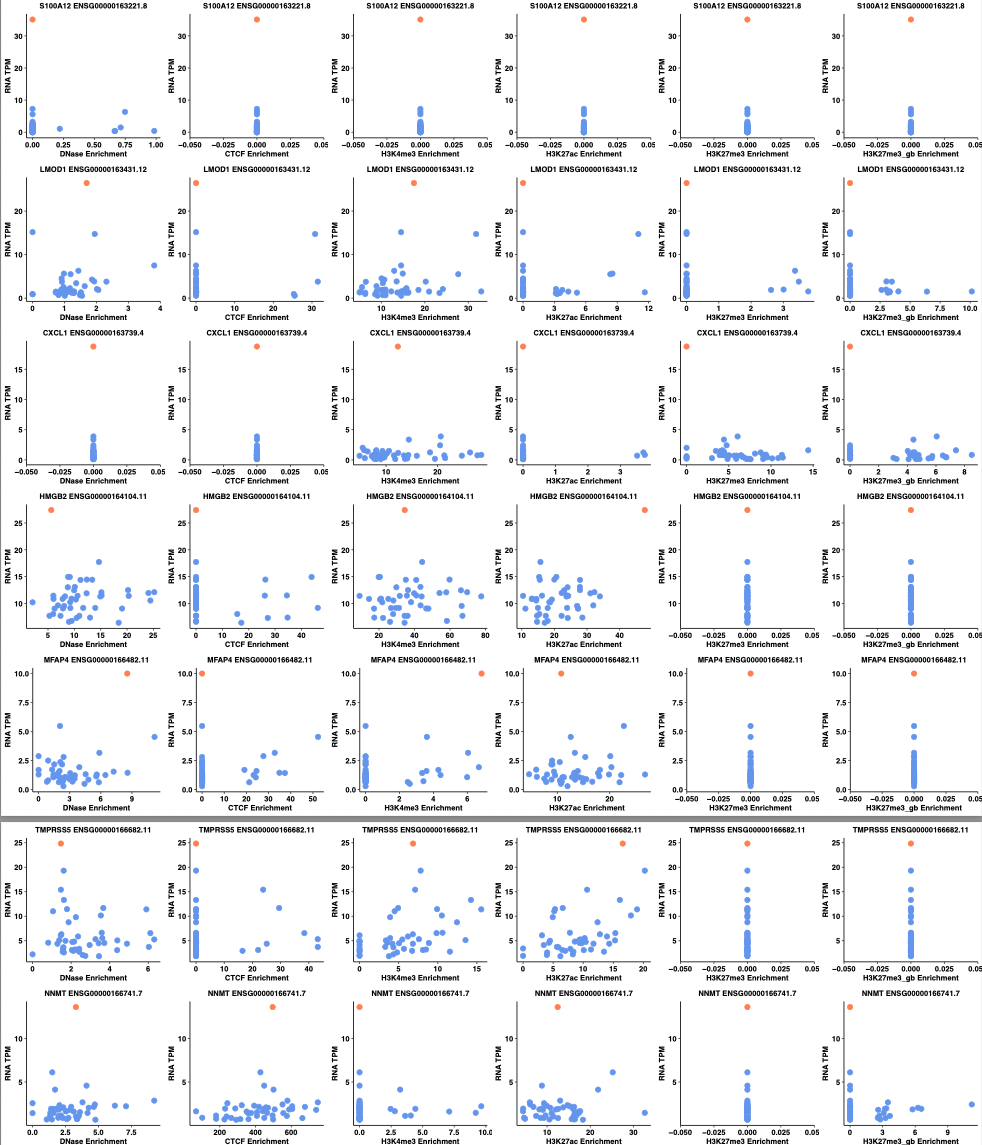


FigureS2 - part17


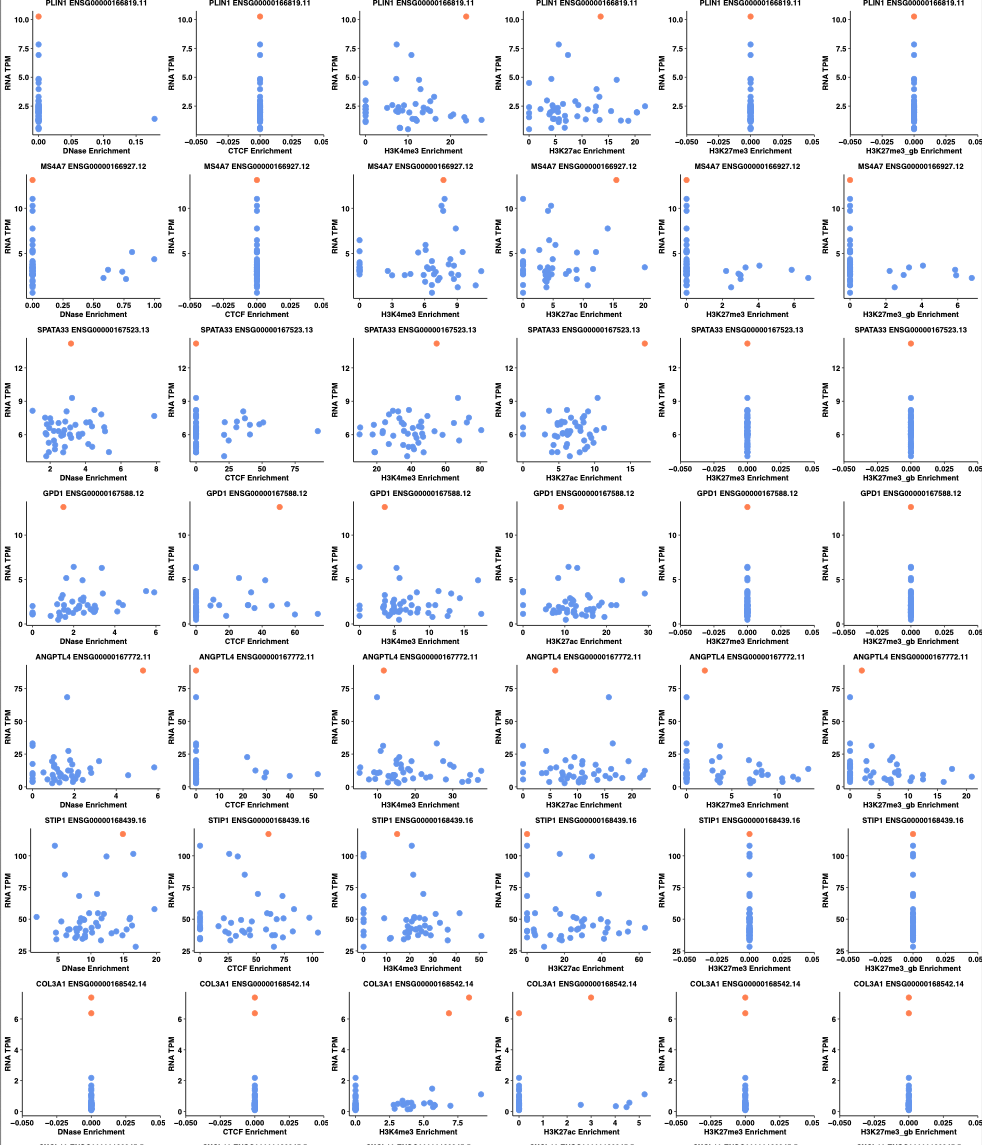


FigureS2 - part18


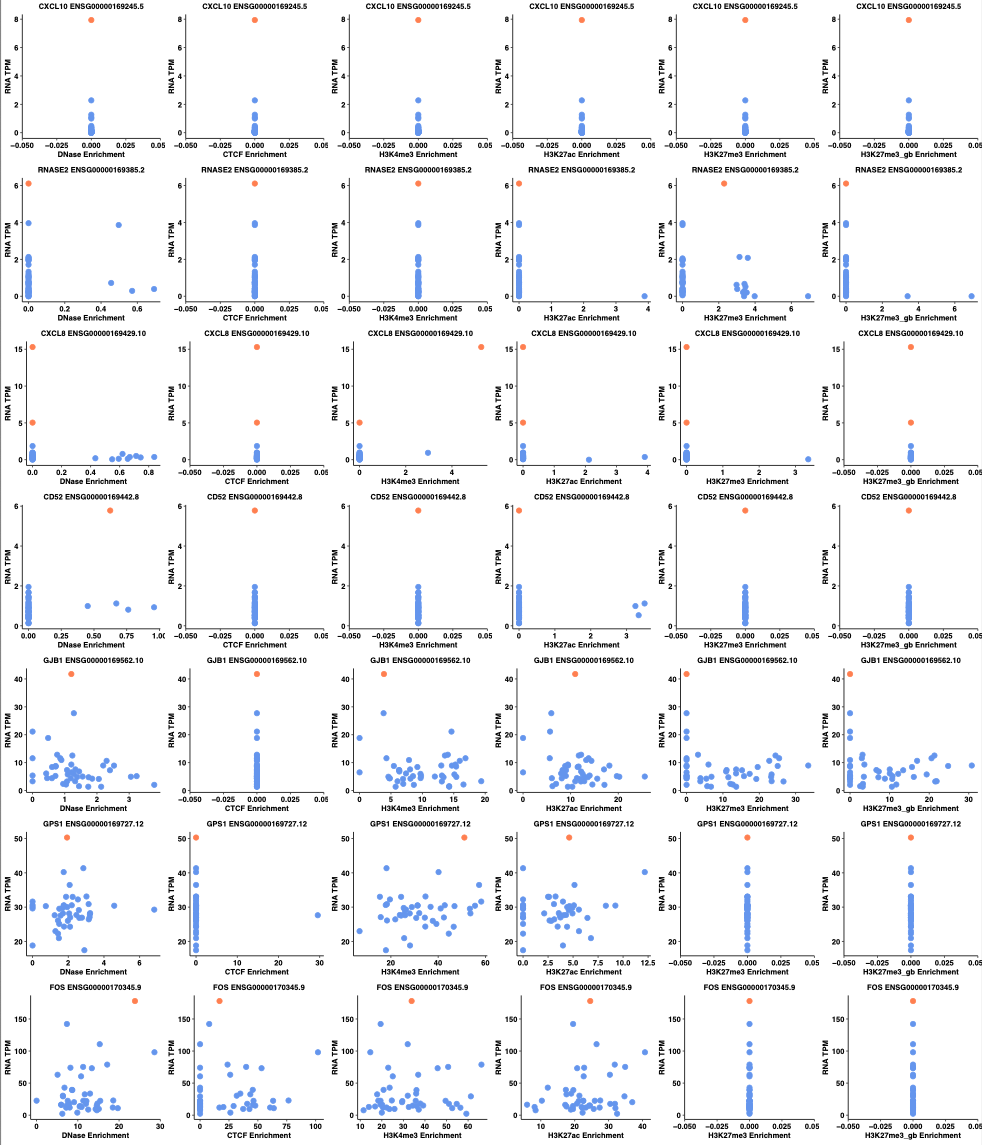


FigureS2 - part19


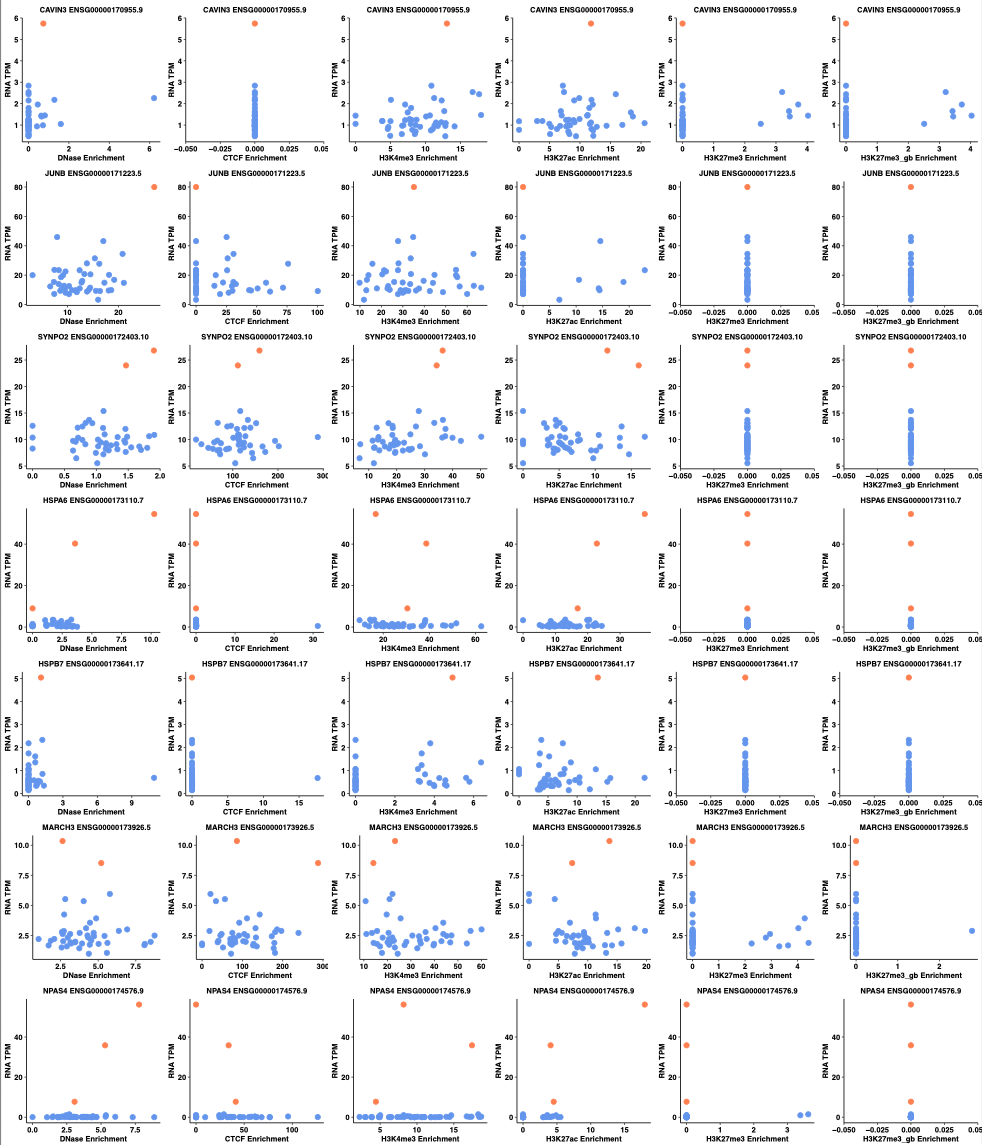


FigureS2 - part20


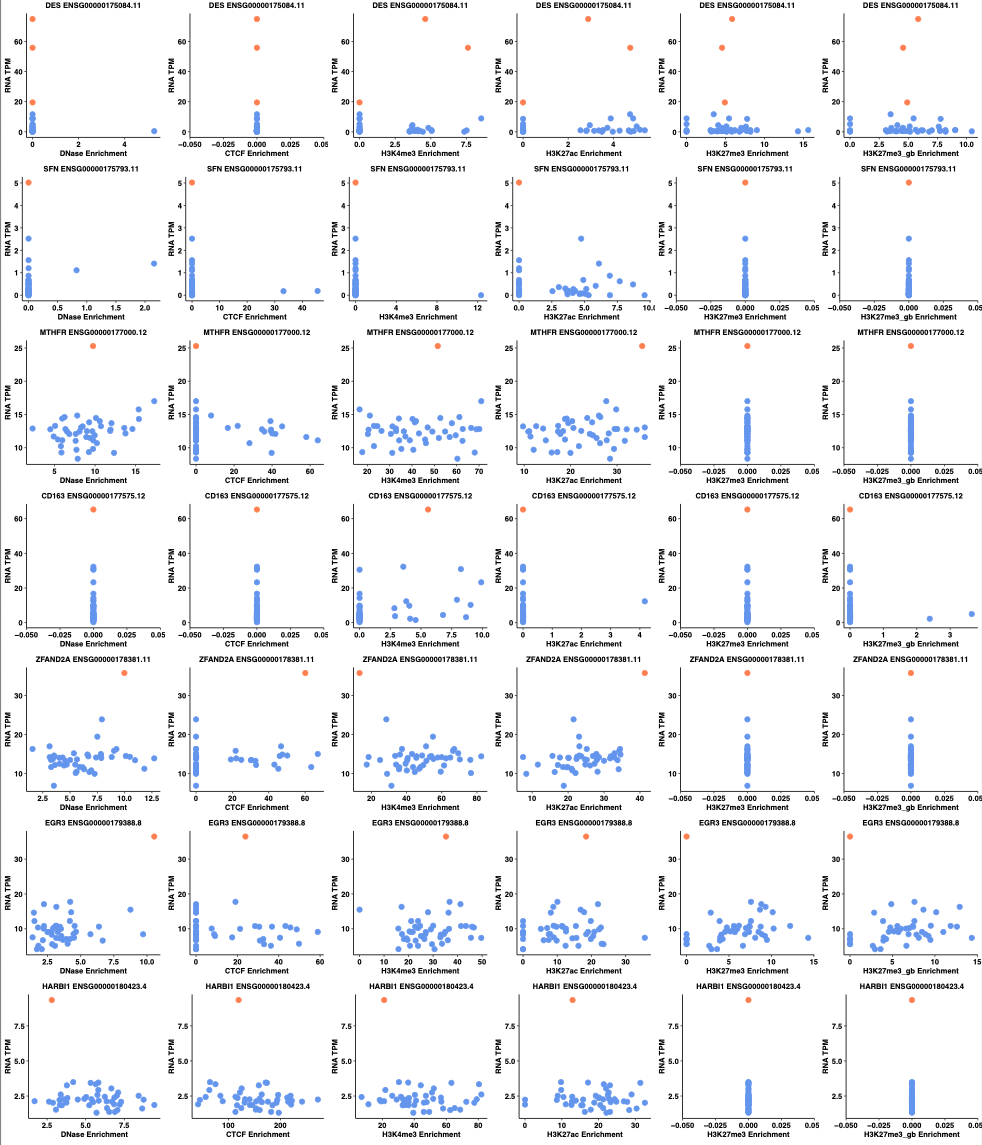


FigureS2 - part21


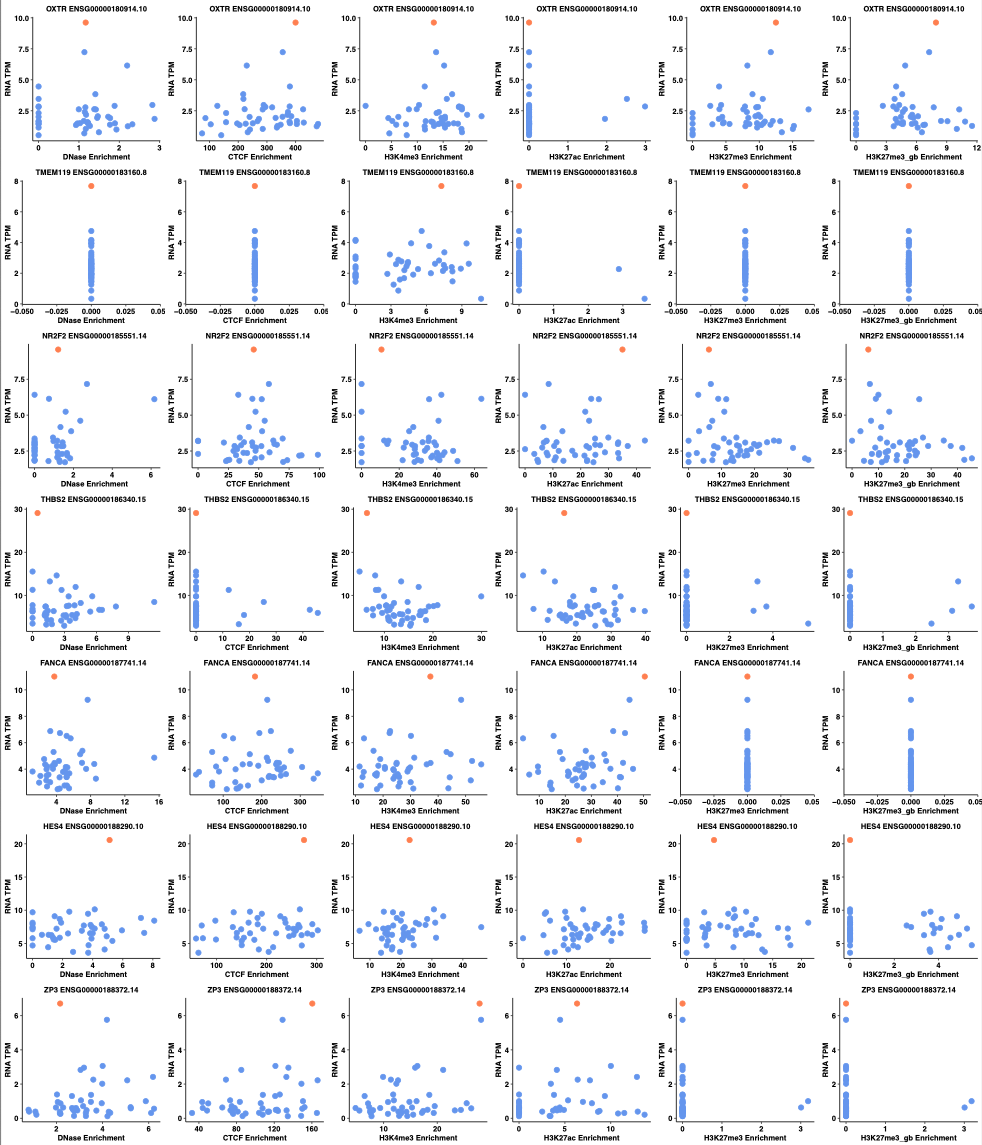


FigureS2 - part22


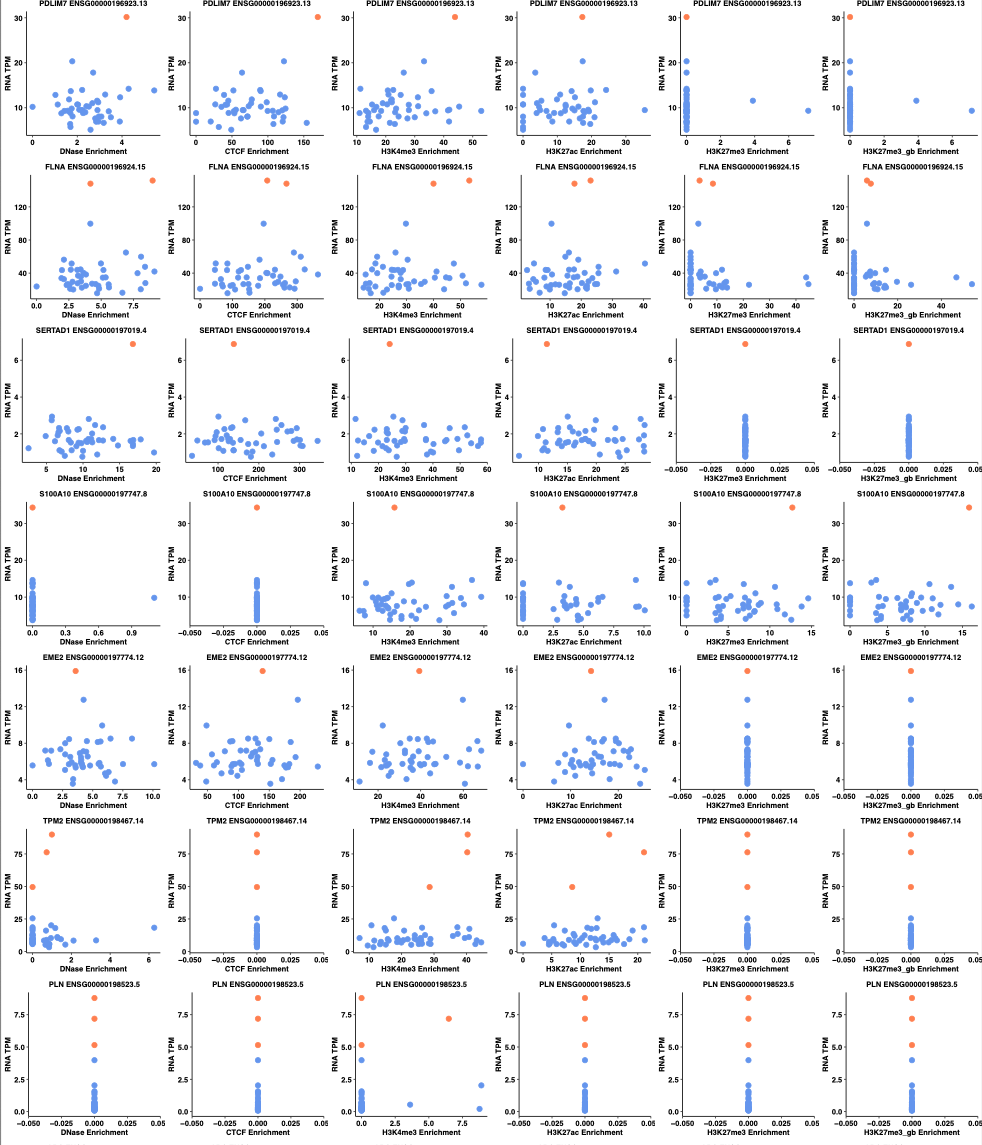


FigureS2 - part23


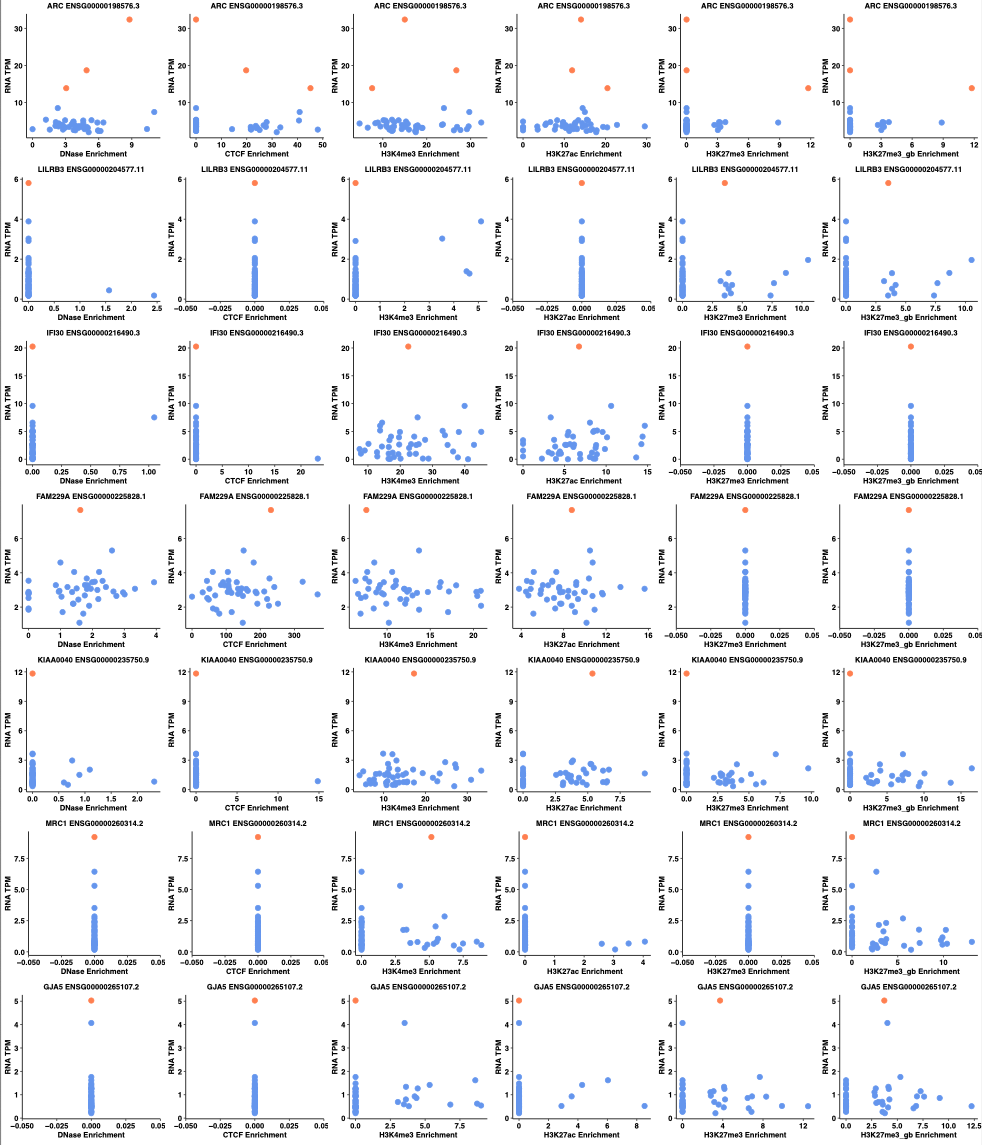


FigureS2 - part24


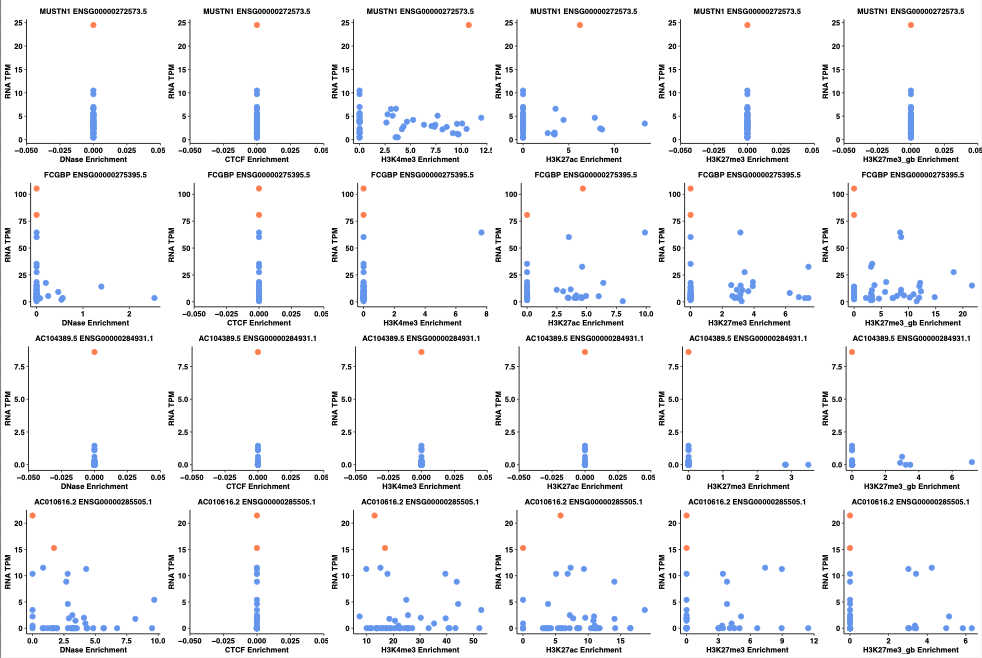

Supplement: Supplementary file 16 — Additional file 16: Figure S2. Visualization of the variance data from Table S14 [file 13059_2025_3709_MOESM16_ESM.docx]
